# Effects of COVID-19 Non-Pharmacological Interventions on Dengue Infection: A Systematic Review and Meta-Analysis

**DOI:** 10.3389/fcimb.2022.892508

**Published:** 2022-05-19

**Authors:** Qin Wu, Shuwen Dong, Xiaokang Li, Boyang Yi, Huan Hu, Zhongmin Guo, Jiahai Lu

**Affiliations:** ^1^ One Health Center of Excellence for Research and Training, School of Public Health, Sun Yat-sen University, Guangzhou, China; ^2^ National Medical Products Administration (NMPA) Key Laboratory for Quality Monitoring and Evaluation of Vaccines and Biological Products, Guangzhou, China; ^3^ Key Laboratory of Tropical Diseases Control, Sun Yat-Sen University, Ministry of Education, Guangzhou, China; ^4^ Sun Yat-Sen College of Medical Science, Sun Yat-sen University, Guangzhou, China; ^5^ Research Institute of Sun Yat-Sen University in Shenzhen, Shenzhen, China; ^6^ Hainan Medical University ' One Health' " Research Center, Hainan Medical University, Hainan, China

**Keywords:** COVID-19, non-pharmacological interventions, dengue incidence, mobility restrictions, meta-analysis

## Abstract

Non-pharmacological interventions (NPIs) implemented during the coronavirus disease 2019 (COVID-19) pandemic have demonstrated significant positive effects on other communicable diseases. Nevertheless, the response for dengue fever has been mixed. To illustrate the real implications of NPIs on dengue transmission and to determine the effective measures for preventing and controlling dengue, we performed a systematic review and meta-analysis of the available global data to summarize the effects comprehensively. We searched Embase, PubMed, and Web of Science in line with PRISMA (Preferred Reporting Items for Systematic Reviews and Meta-Analyses) guidelines from December 31, 2019, to March 30, 2022, for studies of NPI efficacy on dengue infection. We obtained the annual reported dengue cases from highly dengue-endemic countries in 2015–2021 from the European Centre for Disease Prevention and Control to determine the actual change in dengue cases in 2020 and 2021, respectively. A random-effects estimate of the pooled odds was generated with the Mantel-Haenszel method. Between-study heterogeneity was assessed using the inconsistency index (*I^2^*) and subgroup analysis according to country (dengue-endemic or non-endemic) was conducted. This review was registered with PROSPERO (CRD42021291487). A total of 17 articles covering 32 countries or regions were included in the review. Meta-analysis estimated a pooled relative risk of 0.39 (95% CI: 0.28–0.55), and subgroup revealed 0.06 (95% CI: 0.02-0.25) and 0.55 (95% CI: 0.44-0.68) in dengue non-endemic areas and dengue-endemic countries, respectively, in 2020. The majority of highly dengue-endemic countries in Asia and Americas reported 0–100% reductions in dengue cases in 2020 compared to previous years, while some countries (4/20) reported a dramatic increase, resulting in an overall increase of 11%. In contrast, there was an obvious reduction in dengue cases in 2021 in almost all countries (18/20) studied, with an overall 40% reduction rate. The overall effectiveness of NPIs on dengue varied with region and time due to multiple factors, but most countries reported significant reductions. Travel-related interventions demonstrated great effectiveness for reducing imported cases of dengue fever. Internal movement restrictions of constantly varying intensity and range are more likely to mitigate the entire level of dengue transmission by reducing the spread of dengue fever between regions within a country, which is useful for developing a more comprehensive and sustainable strategy for preventing and controlling dengue fever in the future.

## Introduction

Dengue fever (DF) first occurred in Jakarta, Indonesia in 1779. Known as “arthritic fever”, it has evolved into a major public health issue with a higher rate of increase than any other communicable diseases over the past decades, imposing a heavy socioeconomic and disease burden on numerous regions (Wu et al., 2011; Bhatt et al., 2013, GBD, 2016). There are an estimated 400 million DF onset cases per year, which are responsible for 1.1 million disability-adjusted life years globally, and the estimated global economic cost of DF was USD39.3 billion in 2011 (Beatty et al., 2011; Selck et al., 2014; Shepard et al., 2016). Dengue is transmitted between humans *via* mosquitoes of the genus Aedes (mainly Aedes aegypti and A. albopictus), an arthropod common in tropical and subtropical regions (Khetarpal and Khanna, 2016). However, fueled by globalization, climate change, urbanization, and human movement, the geographical distribution of DF is expanding rapidly. Based on updated mosquito distribution maps, Aedes mosquitoes are now found across all continents, placing half the world population at risk of dengue infection and it has been estimated that it will affect more than 6.1 billion people by 2080 (Bhatt et al., 2013; Kraemer et al., 2019; Messina et al., 2019).

Although DF has been detected in more than 100 countries to date, it remains a highly neglected vector-borne disease. In 2019, most countries in Asia and the Americas reported a spike in the number of detected DF cases compared with the same period in previous years. Furthermore, as classical dengue-non-endemic regions, the European Union/European Economic Area (EU/EEA) reported a 2019 dengue infection rate that was 2.5 times higher than that in 2018 (Control, E. C. F. D. P. A., 2021). Earlier in 2020, the World Health Organization (WHO) listed DF among 10 diseases that were potential threats for 2019 (Organization, W. H., 2020a). Currently, there is no efficient vaccine and specific treatment available for DF, and mosquito vector control is the most dominant and crucial measure for governments to prevent and control DF even though it is mitigative. Given the extremely reproductive property of mosquitoes, it is apparent that mosquito control must be a long-term task without any respite and that developing more comprehensive and sustainable strategies to stop the spread of DF is imperative.

In early December 2019, a pneumonia of unknown origin was first reported in the city of Wuhan in Hubei province, China (To et al., 2021). Subsequently, it spread worldwide within weeks. On February 11, 2020, the WHO officially named the pneumonia coronavirus disease 2019 (COVID-19) and declared the infectious disease a global pandemic (Huang et al., 2020). The COVID-19 pandemic is still spreading at an unrelenting pace in most countries. In response to this pandemic, central governments worldwide have enforced a series of nonpharmaceutical interventions (NPIs), public health measures aimed at suppressing infectious disease transmission. These crucial NPIs are outlined as international movement restrictions (border/travel restrictions), quarantine and isolation, internal movement restrictions or physical distancing (large-scale lockdowns, social distancing), community management, face mask usage, and personal hygiene and emergency investments (Hale et al., 2020) . Early in the pandemic, NPIs were implemented strictly throughout most countries, particularly the essential NPIs aimed at “preventing diffusion inside and importing outside” due to the lack of a reliable antidote for this unprecedented emerging infectious disease. With COVID-19 vaccines and accumulated prevention and control experience, countries began to relax these measures in an orderly manner.

Nonetheless, NPIs continue to play a critical role in containing the COVID-19 pandemic. Such NPIs exert positive effects for containing and mitigating COVID-19 transmission and have also generated potential impacts on the prevalence of other diseases, especially infectious diseases. Currently, a number of researchers have demonstrated that NPIs implemented during the COVID-19 pandemic have led to substantial reductions in the infectious disease burden in almost all notification categories under routine national surveillance. Nevertheless, their impact on DF is mixed, particularly for dengue-endemic regions (Brady and Wilder-Smith, 2021). As far as we know, there is no systematic reviews being carried out to assess the impact of NPIs on DF infection.

Hence, to illustrate the real implications of NPIs on dengue transmission and to determine the effective measures for dengue prevention and control, we selected all qualified articles focusing on assessing the impacts of NPIs on dengue infection and collected the available public data on dengue cases to analyze their effects from a more comprehensive perspective.

## Methods

This study was conducted and reported in line with the Preferred Reporting Items for Systematic Reviews and Meta-Analyses (PRISMA) guidelines (Liberati et al., 2009). The review is registered with PROSPERO (CRD42021291487), on which the study protocol is available.

### Literature and Public Data Search

We retrieved articles published through December 31, 2019, to March 30, 2022, from Embase, PubMed, and Web of Science. The keywords were identified by searching the Medical Subject Headings (MeSH) database. The search terms were: (“dengue” OR “DENF” OR “breakbone” OR “break-bone” OR “arboviruses” OR “arboviral” OR “arbovirus” OR “mosquito-borne” OR “arthropod-borne”) AND (“COVID-19” OR “2019-nCoV” OR “Coronavirus disease-19” OR “2019 novel coronavirus disease” OR “COVID 19” OR “SARS-CoV-2” OR “SARS CoV 2”) and were restricted to the article title and abstract. The search strategy used in PubMed is presented in **Supplementary Text 1**. Additional relevant papers were manually searched from the reference lists of the included publications. We also obtained the published data on annual reported dengue cases during 2015–2021 from the European Centre for Disease Prevention and Control (ECDC).

### Inclusion and Exclusion Criteria

Studies on the impact of COVID-19 NPIs on DF incidence rates were included in this review. The inclusion criteria were: 1) focused on humans; 2) the type of study is prevalence study, cohort study, or case-control study; 3) reported and compared DF case numbers or incidence before and during the COVID-19 pandemic, or illustrated the effects of COVID-19 on dengue infection with a quantitative analysis. The exclusion criteria were: 1) duplicate articles (including both study site and analytic method duplication); 2) reviews and systematic reviews, conference abstracts, dispatches, short reports, short communications and editorial letters; 3) data without clear sources or no detailed data on dengue infection; 4) no comparison group or other unrelated study design; 5) abstracts or full-text not available. No language restrictions were applied and all articles not in English were translated and included or excluded based on the above criteria.

### Study Selection and Data Extraction

The titles and abstracts of all retrieved citations were imported and cataloged in Endnote X9 and duplicates were removed. Two authors (QW, SWD) screened the articles independently by reading the titles and abstracts. Any conflicts were discussed, with adjudication by a third reviewer (XKL) if necessary. The full texts of all potentially eligible studies were retrieved for further assessment. Two reviewers (QW, SWD) extracted the data from the final eligible articles independently and in duplicate using a standard information collection table and resolved disagreements by consensus. For studies that performed stratified analysis of response levels, the response duration and results at different levels were recorded to assess the effects of the leading NPIs implemented for each response level on dengue infection.

### Quality Assessment

Two reviewers (QW, XKY) assessed the quality and risk of bias of each included study independently according to the Newcastle-Ottawa Scale (NOS), comprising of the selection of study participant groups (four stars), the comparability of study groups (two stars), and the ascertainment of outcome (three stars). The NOS was modified according to the study types to enable better appraisal of the study quality (**Supplementary Table 1**). The detailed criteria for the NOS items are as follows: 1)Selection of non-exposed: the incidence of the non-exposed can represent the history incidence level (one star); 2) Ascertainment of exposure: the article detailed the timing of NPIs for COVID-19(one star); 3) Demonstration that outcome of interest was not present at start of study: the non-exposed group was not exposed to NPIs for COVID-19 (one star); 4) Comparable for onset seasons: incidence rates in the exposed and non-exposed groups were compared over the same period (one star); 5) Sufficiently long follow-up for outcomes to occur: the observation time of the exposure group included the peak of the dengue epidemic (one star); 6) Adequacy of follow-up: the influencing factors of underreporting were the same in the exposed and non-exposed groups (one star). Studies with a full rating in at least two categories of selection, comparability, or outcome assessment were considered to have low risk of bias (Gao et al., 2018). Begg’s test were used to identify the potential publication bias. Finally, GRADE was performed to assess the quality of evidence of the analyzed outcomes following the guidelines of Cochrane institution by using GRADEprofiler software (version 3.6).

### Data Analysis

Different meta-analyses were performed on the included articles based on their data analysis methods. Articles without detailed case numbers in the control and exposed groups or that did not report the confidence interval (CI) value for study indicators were excluded from the meta-analysis. A random-effects estimate of the pooled odds with the 95% CI of the outcome was generated with the Mantel-Haenszel method. Between-study heterogeneity was explored using the inconsistency index (*I^2^*) statistic. Subgroup analysis was performed based on whether the study site was dengue-endemic or non-endemic. All data analyses were performed using Stata 12. The mean DF onset cases before the COVID-19 pandemic was calculated based on the ECDC data. Subsequently, we determined the change rate of DF cases during the COVID-19 pandemic as compared to the previous onset level.

## Results

### Study Characteristics

**Figure 1:**
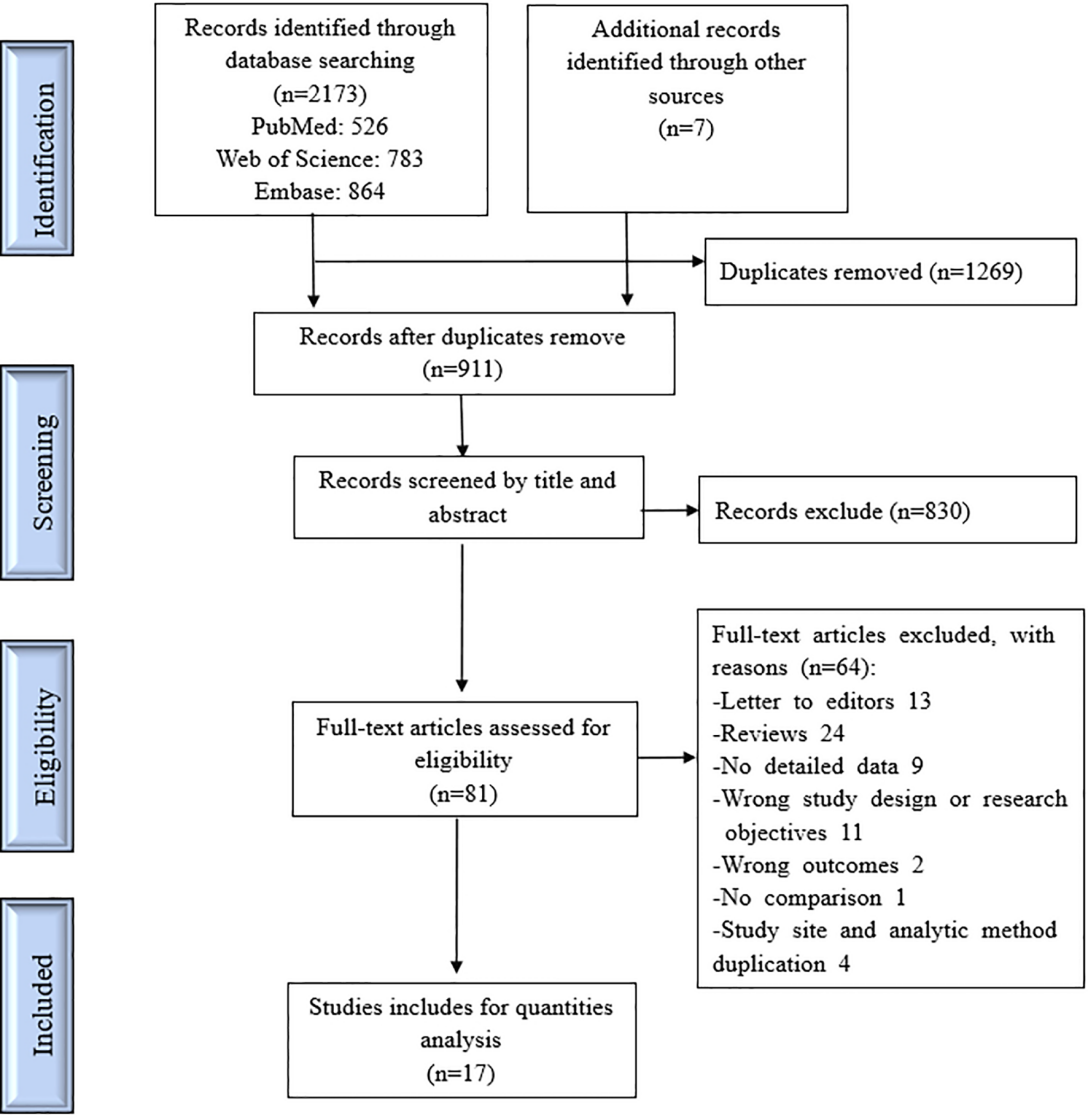
Flow diagram of publication selection process.

Of 2173 studies identified from the electronic databases and the reference lists of included publications, 81 were eligible for full-text review and 17 met the inclusion criteria for the systematic review (**Figure 1**). **Supplementary Table 2** lists the excluded articles with specific reasons for exclusion as determined *via* full-text review. Our review covered 32 study countries and regions: Asia, Europe, Oceania, and America, while the Southeast Asia and Latin America regions were mainly research sites. In eleven articles, the study site was in a dengue-endemic country. All DF cases in those studies were obtained from the corresponding disease surveillance systems and the study time for NPIs was limited to 2020. The methods in the included studies for evaluating the effect of COVID-19 NPIs on dengue infection were roughly divided into A, B, C according to their statistical analysis technique. **Supplementary Table 3** lists their detailed definitions. The modified NOS demonstrated that the quality of those studies was mainly indicated by 6–9 points. **Supplementary Table 3** contains the detailed data extraction information for all included studies. **Table 1** summarizes the basic characteristics of the included articles.

**Table 1:** Characteristics of studies included in the systematic review and meta-analysis.

Research	Study site	Endemic or not	Data collection period		Analytic method^a^	Cases		Population size/million	EI	Result	Quality*
			Pre-pandemic	pandemic		Control group	Exposed group				
Steffen et al., 2020	Switzerland	NO	Weeks 15-26 in 2016-2019	Weeks 15-26 in 2020	A	38	4	8.637	RC	89.5%	6
Rahim et al, 2021	Peninsular Malaysia	YES	Weeks 10-11 in 2020	Weeks 12-17 in 2020	A	7268	3747	32.730	RC	48.45%	4
Niriella et al., 2021	Sri Lanka	YES	April to June 2019	April to June 2020	A	13249	3492	22.000	RC	73.6%	4
Li et al., 2021a	Thailand	YES	2013-2019	2020	A	68739	50042	69.800	RC	27.2%	7
	Viet Nam	YES	2013-2019	2020	A	137328	121398	97.339	RC	11.6%
	Laos	YES	2013-2019	2020	A	16712	7554	7.231	RC	54.8%
	Yunnan	YES	2013-2019	2020	A	2241	260	47.222	RC	88.4%
Bright et al., 2020	Australia	NO	January to June 2015-2019	January to June 2020	A	918	192	25.700	RC	79.0%	6
Lai, 2021	Taiwan	NO	January and September 2019	January and September 2020	A	408	59	23.561	RC	85.5%	6
Xiao et al., 2021	Guangdong,China	NO	2015-2019	2020	B	-	-	-	RR	0.007(0.004,0.009)	8
Lu et al., 2021	Indonesia	YES	2015-2019	2020	B		–	–	RR	1.06(1.05,1.07)	7
	Australia	NO	2015-2019	2020	B	–	–	–	RR	0.14(0.12.0.16)	
	Belize	YES	2014-2019	2020	B	-	-	-	RR	1.77(0.73, 1.94)	
	Bolivia	YES	2014-2019	2020	B	-	-	-	RR	1.42(0.32,4.29)	
	Brazil	YES	2014-2019	2020	B	-	-	-	RR	13.25(1.11,42.54)	
	Colombia	YES	2014-2019	2020	B	-	-	-	RR	0.61(0.20,1.48)	
	Costa Rica	YES	2014-2019	2020	B	-	-	-	RR	1.26(0.40,3.08)	
	Dominican Republic	YES	2014-2019	2020	B	-	-	-	RR	0.07(0.02,0.18)	
	Ecuador	YES	2014-2019	2020	B	-	-	-	RR	0.51(0.14,1.33)	
	EL Salvador	YES	2014-2019	2020	B	-	-	-	RR	0.27(0.08,0.68)	
Chen et al., 2022	Guatemala	YES	2014-2019	2020	B	-	-	-	RR	0.13(0.04,0.23)	9
	Honduras	YES	2014-2019	2020	B	-	-	-	RR	1.18(0.39,2.77)	
	Jamaica	YES	2014-2019	2020	B	-	-	-	RR	0.05(0.01,0.13)	
	Mexico	YES	2014-2019	2020	B	-	-	-	RR	0.76(0.20,2.09)	
	Nicaragua	YES	2014-2019	2020	B	-	-	-	RR	3.08(0.97,7.70)	
	Panama	YES	2014-2019	2020	B	-	-	-	RR	0.19(0.06,0.44)	
	Peru	YES	2014-2019	2020	B	-	-	-	RR	2.01(0.60,5.38)	
	Venezuela	YES	2014-2019	2020	B	-	-	-	RR	0.10(0.03,0.25)	
	Cambodia	YES	2014-2019	2020	B	-	-	-	RR	0.18(0.05,0.46)	
	Laos	YES	2014-2019	2020	B	-	-	-	RR	0.58(0.17,1.43)	
	Malaysia	YES	2014-2019	2020	B	-	-	-	RR	0.76(0.22,1.96)	
	Philippines	YES	2014-2019	2020	B	-	-	-	RR	0.15(0.04,0.38)	
	Singapore	YES	2014-2019	2020	B	-	-	-	RR	2.21(0.65,5.49)	
	Thailand	YES	2014-2019	2020	B	-	-	-	RR	0.34(0.10,0.86)	
	Vietnam	YES	2014-2019	2020	B	-	-	-	RR	0.66(0.18,1.66)	
Ullrich et al., 2021	Germany	NO	Weeks 10-32 in 2016-2019	Weeks 10-32 in 2020	C	–	–	–	RR	0.249(0.205,0.301)	7
Lim et al., 2020	Thailand	YES	2019	2020	C	-	-	-	RR	1.537(1.061,2.247)	7
	Malaysia	YES	2019	2020	C	-	-	-	RR	0.996(0.982,1.012)
	Singapore	YES	2019	2020	C	-	-	-	RR	1.037(0.891,1.206)
Plasencia-Dueñas et al., 2021	Peru	YES	2018-2019	2020	C	–	–	–	RR	3.93(3.87-3.99)	7
Liyanage et al., 2021	Sri Lanka	YES	January to March in 2015-2020	April to June 2020	C	-	-	-	RR	0.12(0.08-0.17)	7
Conceição et al., 2021	Sao-Paulo, Brazil	YES	January to February 2020	February to August 2020	C	–	–	–	RR	0.909(0.858,0.962)	6
Lim et al., 2021a	Singapore ^b^	YES	2003-2019	2020	C	-	-	-	RR	1.372(1.199,1.498)	7
Lim et al., 2021b	Singapore ^c^	YES	January 2013 to April 2020	April to May 2020	C	–	–	–	RR	0.315	8
	Singapore ^d^	YES	January 2013 to April 2020	April to May 2020	C	–	–	–	RR	1.635
Li et al., 2021a	Yunnan, China	NO	2013-2019	2020	C	-	-	-	RR	0.052	7

^‘a’^: A,B,C represent single-arm design, time series analysis and regression analysis respectively. ^‘b’^: The study population is aged 5-65. ^‘c’^: The study population was migrant workers. ^‘d’^: The study population was general workers aged 20-65. “-”: The data is unavailable and is not necessary for meta-analysis by using “Effect/CI”.

‘*’: The max score for quality is 9. “EI”, Effect indicator; “RC”; Relative change (%); “RR”, Relative risk.

### The Effects of COVID-19 NPIs on Dengue Infection

**Figure 2:**
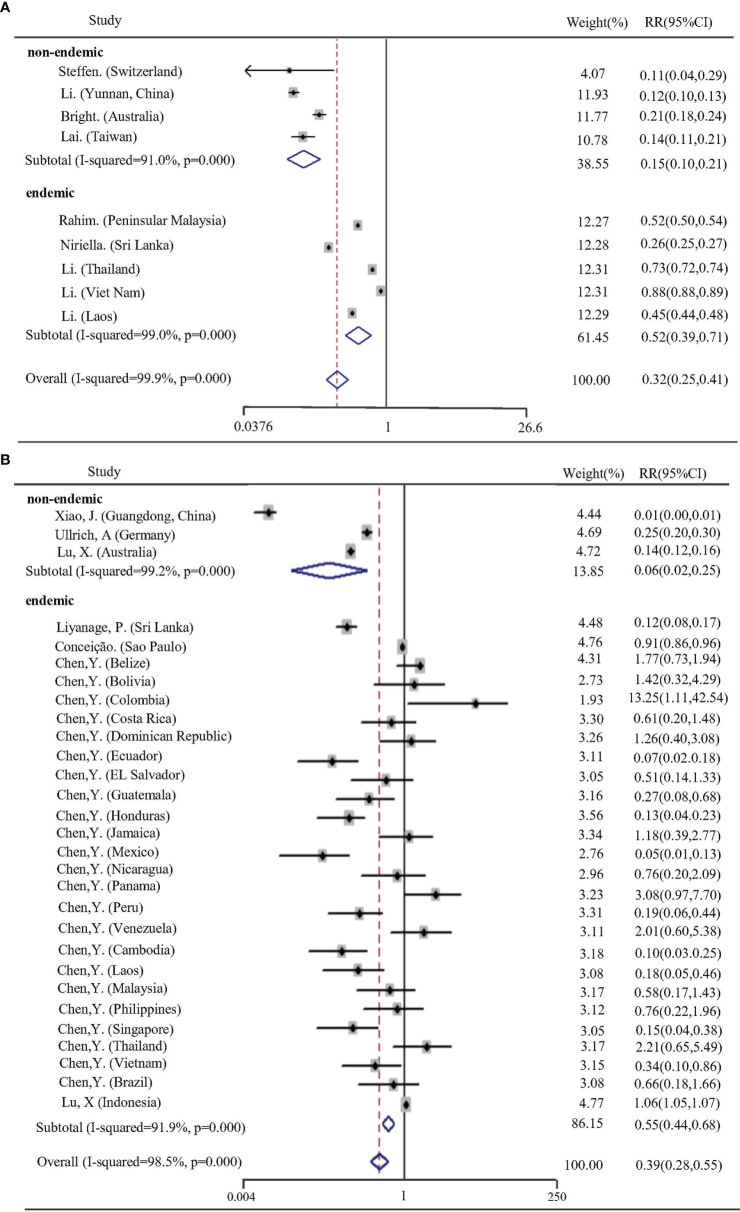
Forest plot of pooled RRs for the effects of NPIs on dengue infection in 2020. **(A, B)** correspond to different statistical analysis groupings described above. RRs are random-effects estimates calculated by Mantel-Haenszel method.

Six studies (Bright et al., 2020; Lai et al., 2021; Li et al., 2021a; Niriella et al., 2021; Rahim et al., 2021; Steffen et al., 2020) reporting nine effect estimates and using analytic methods classified as ‘A’ were used for meta-analysis in a count data manner. The total pooled RR was 0.32 (95% CI: 0.25–0.41). Random-effects analysis revealed significantly high heterogeneity (*I^2^* = 99.9%, p < 0.0001). Meta-regression applied to explore the potential source of heterogeneity determined that only the “endemicity” difference of dengue was significant, which explained 51.88% between-study variance (**Supplementary Text 2**). Subgroup analysis revealed that the pooled RR in non-endemic areas (RR = 0.15, 95% CI: 0.10–0.21) was lower than that of endemic countries (RR = 0.52, 95% CI: 0.39–0.71) (**Figure 2A**). Twenty-nine effect indicator values from six studies (with analytic methods classified as BC) (Ullrich et al., 2021; Liyanage et al., 2021; Conceição et al., 2021; Xiao et al., 2021; Lu et al., 2021; Chen et al., 2022)were pooled to reveal an RR of 0.39 (95% CI: 0.28–0.55) through the effect/CI data setting. The pooled estimate in endemic group was nine times higher than that in non-endemic group, a pooled RR 0.06 (95% CI: 0.02-0.25) and 0.55 (95% CI: 0.44-0.68) respectively, in 2020 (**Figure 2B**).

Two studies by Lim et al. (Lim et al., 2021b; Lim et al., 2021a) were not used to merge effects, as their study population was workers and both study sites were in Singapore. In Singapore, social distancing measures significantly increased the risk of dengue infection in general workers and students while the quarantine measures against migrant workers demonstrated a positive effect for reducing dengue infection. In addition, the two articles by Lim et al. and Plasencia et al. (Lim et al., 2020; Plasencia-Dueñas et al., 2021) with data collection period of less than two years in non-exposed group(COVID-19 pre-pandemic) were selected for comparison with the article that had the same study site and analytic methods but a longer data collection period in non-exposed group. A subgroup meta-analysis revealed that the pooled RR value in the study with less than two years of observation in the control group was overestimated (**Supplementary Figure 1**).

Begg’s test undetected the publication bias (p=0.149), but there were some the points on the outside of the funnel plot (**Supplementary Figure 2**), indicating significant heterogeneity between studies. GRADE assessment revealed a “Moderate” quality grade of evidence in the group of “BC”, while a “Very low” quality grade in “A” group (**Supplementary Figure 3**).

### Impacts of COVID-19 NPIs at Different Stringency Levels on Dengue Infection

Other than China, the countries included in our review successively initiated high-stringency responses against COVID-19 since early March 2020, and began to lower the stringency levels, mainly in restricting human behavior, after almost 2–3 months (**Supplementary Text 3**). In this review, only two articles (Xiao et al., 2021; Ullrich et al., 2021), in which both study sites were in DF non-endemic areas, studied the effects of COVID-19 NPIs on dengue infection based on the varied degrees of stringency. Both studies reported an increased rate of reduction in the number of DF cases as the response levels decreased (**Supplementary Table 5**).

**Figure 3:**
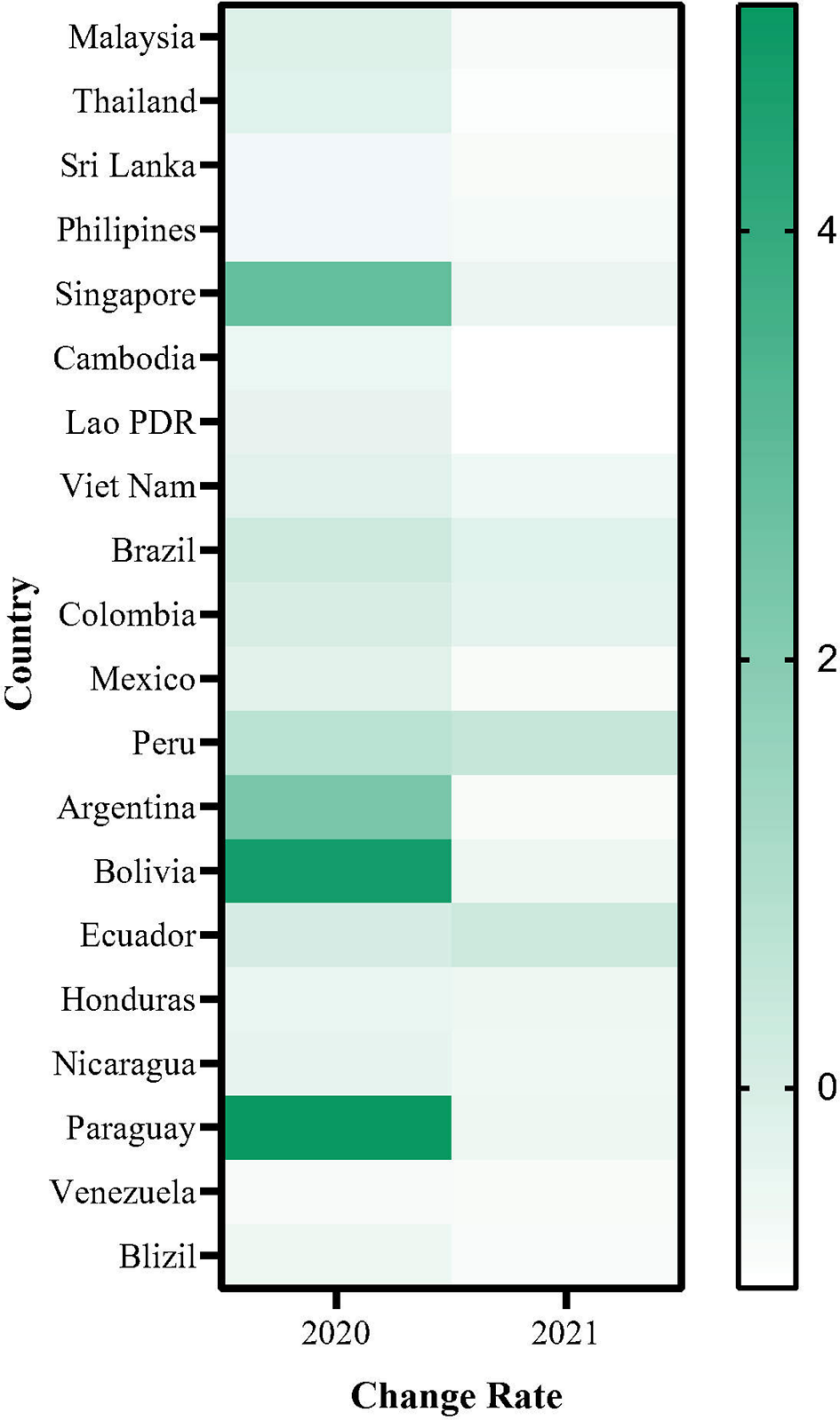
The change rate of notified dengue case during COVID-19 in Asia and Americas. Change rate= (the number of dengue cases in 2020 or 2021 - the average number of dengue cases in 2015-2019)/ (the average number of dengue cases in 2015-2019).

The total DF cases in the Americas in 2020 was reduced by 27% compared to that in 2019 but was 30% higher than the average in 2015–2019. Subsequently, a 30% reduction rate was reported for 2021 as compared to the average (**Supplementary Table 4**). Further, 20 countries in Asia and the Americas, the most highly endemic areas worldwide, were selected to demonstrate the relative change in dengue cases during the COVID-19 pandemic as compared with the average in 2015–2019. **Figure 3** shows that 65% (13/20) of these countries reported a 0–100% reduction in dengue cases in 2020 compared to previous years, while some countries (4/20) reported a dramatic increase, resulting in an overall increase of 11%. In contrast, almost all countries (18/20) observed an obvious reduction in dengue cases in 2021, with an overall 40% reduction rate.

## Discussion

Our systematic review summarizes the available global published studies and was aimed at estimating the effects of the NPIs implemented during the COVID-19 pandemic and analyzing the influence of specific NPIs on DF infection. Following the initial imposition of NPIs in China, almost all countries and regions began to implement common measures during the first wave of the pandemic, with the highest stringency recorded in mid-April 2020 and spanning 2–3 months, followed by gradually relaxed stringency globally (Liu et al., 2021). Overall, NPIs were significantly less stringent in 2021 than in 2020, where the changes were mainly in terms of restricting people’s movements, but measures such as recommendations to stay home, restrictions on internal movement, and screening or quarantine for international travelers remained, although at lower stringency (Tracker., O. C.-G., 2022) . The unprecedented scale and intensity of the COVID-19 NPIs worldwide exacted a huge cost and have provided a unique opportunity for evaluating their effectiveness on the circulation of DF from a global perspective. And the distinct variation in implementation intensity across countries and over time enabled dissection of the effect of certain key variables on disease *via* cross-sectional and longitudinal comparisons.

In an effort to ensure as robust an identification of the effect of the NPIs as possible and to avoid artifacts of other factors that affected dengue transmission during the COVID-19 pandemic, standardized or multivariate analysis methods, such as time series models or regression models, were used for the data analysis to control known confounders in our included studies. With this in mind, the effect values of our meta-analysis were pooled respectively according to the analysis method (A and BC) to reduce the apparent existing inter-study heterogeneity from the statistical approach. Although we excluded the necessary heterogeneity for statistical bias, significant heterogeneity between studies was nevertheless present in our meta-analysis and could not be entirely explained by subgroup differences according to country. Coincidentally, a similarly high level of heterogeneity for dengue prevalence was documented by Eltom et al. and Li et al. (Eltom et al., 2021; Li et al., 2021b). Clearly, the heterogeneity of DF epidemic status assessment is multifactorial due to the geographical inequality of dengue epidemics and the diverse emphasis of dengue prevention and control between countries. Therefore, it may be advisable to consider regions with common epidemiological conditions (e.g., low resources, poor disease surveillance systems, highly endemic for dengue, common cultural background) as an integrated approach for implementing both DF disease burden evaluation and prevention and control strategy development.

Our meta-analysis showed a large decrease in DF cases during the COVID-19 pandemic as compared with pre-pandemic at the global level, and the dengue-non-endemic regions persistently presented a dramatic reduction rate. Although the total DF cases in the Americas in 2020 were higher than the average in 2015–2019, positive effects of NPIs on reducing DF incidence were rendered after controlling for climatic and time confounders. It appeared that the assessment of NPI’ efficacy on DF incidence was influenced by a number of factors, from the choice of control group to other natural or social factors. In 2020, a few dengue-endemic countries recorded a significant increase in DF cases. Intriguingly, the countries that did not experience a substantial rise in DF cases in 2019 were more likely to exhibit a spike in 2020, and vice versa. In the epidemiological sense, the dominant reasons for this phenomenon are enhanced population immunity only against the same dengue virus serotype and rigorous mosquito control activities after dengue outbreaks (Reiner et al., 2014). That is, in some dengue-endemic countries, high population susceptibility and neglected mosquito control campaigns may promote a high DF incidence and obscure the real effects of NPIs on DF infection. Combined with the wide reduction in DF cases in Asia and the Americas in 2021, it revealed the overall positive efficacy of NPIs on dengue infection in dengue-endemic areas, albeit not as significantly as that in dengue-non-endemic areas. Compared to 2020, the DF reduction rate declined by a larger magnitude in 2021, demonstrating normalizing NPI’ implementation at a less stringent level was more beneficial to DF control.

Dengue virus transmission is multifactorial and mainly includes human and mosquito movement, mosquito abundance (affected by climatic and environmental factors), human–mosquito–virus interactions, human host immunity, and virus genotype (Wearing and Rohani, 2006; Oidtman et al., 2019). Facilitating human transnational movement, globalization is the most critical and fundamental contributor to DF spread from endemic areas to non-endemic areas. In non-endemic areas, dengue typically starts with a travel-associated introduction, followed by local cases (Pagani et al., 2020). Using the EU/EEA as an example, where DF is not endemic, a vast majority of reported DF cases are always travel-associated and the seasonal pattern of such cases is highly consistent with the tourist season (Control, E. C. F. D. P. A., 2019) . Aiming to containing the spread of COVID-19 (Lemey et al., 2020), 57% of destinations (countries and territories) in the World Tourism Organization introduced travel restrictions by March 16, 2020, increasing to 100% by April 20, 2020 (Organization, W. T., 2020b) . Subsequently in the same year, tourism decreased from 58% to 78% (Organization, W. T., 2020c) . The unprecedented travel restrictions largely limited human international movement, including potential human or even mosquito carriers of infection. At the same time, the restrictions discouraged the sick, particularly people with fever, from traveling. That is, travel restrictions and border bans demonstrated a positive effect in preventing imported dengue cases from high-endemic areas, reducing the risk of local spread effectively. Adversely, the policies have severely affected the tourism industry, resulting in the global economic loss of an estimated USD400 billion and placing millions of jobs at risk each month (Mallapaty, 2021). To achieve economic trade-off, most countries eased the travel restrictions to various extents, ranging from narrowing the scope of restriction to adjusting the policies for human entry to the country (Le et al., 2021). Although reopening international borders and easing travel restrictions increased the risk of importing dengue infection, the continued quarantine measures against returning residents and tourists from COVID-19 epidemic regions were also effective. A 14-day mandatory quarantine in a centralized isolation area was often adopted, which was sufficient for detecting symptomatic dengue cases early and for avoiding the spread of latent dengue cases in the community environment, as DF has an incubation period of 3–7 days before the abrupt onset of symptoms (Wu et al., 2011). Faced with international travelers, some countries adopted shorter mandatory quarantine durations or only required for a negative new crown nucleic acid test report within certain hours of entry (Brigade, 2020).

These adjusted measures greatly reduced the side effects of quarantine and isolation on mental health, the economy, and ethics, which may be an appropriate strategy for balancing economic development and civil liberties under the new normal of COVID-19. Overall, travel-related NPIs have been greatly beneficial in curbing DF importation, especially for dengue-non-endemic areas, without taking into account the economic impact. Nevertheless, the experience of COVID-19 containment in reducing the number of imported cases, particularly the adjusted measures, provides a valuable lesson. For example, in the future, travelers and returnees from dengue-endemic areas as identified by the relevant authorities can be requested to provide a proof of dengue virus negative nucleic acid or negative antigen test as their health pass to enter the country. This would not only decrease the risk of importing dengue cases but would also aid the detection of unapparent and asymptomatic dengue cases in the population to bridge the gaps in existing dengue disease surveillance systems.

Physical distancing measures, such as bans on gatherings and closing schools and workplaces, encourage people to stay at home and are principal countermeasures for alleviating COVID-19 spread with unquestionably remarkable effects. Nonetheless, these measures have a complex impact on DF transmission. The implementation intensity of these measures is adjusted discontinuously based on the number of COVID-19 cases in the local area, and range from complete lockdown to partial movement restrictions at city-wide or national level. Such measures, especially total lockdowns in the early days of the epidemic, dramatically reduced internal movement and altered daily routine movement patterns, and featured increased household movement and decreased workplace movement. The main vector of DF, A. aegypti, a daytime feeder, is compatible in urban habitats and breeds predominantly in human-made water-logged containers, persisting in residential areas. On one hand, the increased time at home may heighten the risk of intra-household transmission, given the increased number of unique individuals bitten by a single mosquito under the condition of being highly clustered in a residential location (Cavany et al., 2021). In addition, some researchers believe that the strict social distancing measures disrupted routine Aedes surveillance vector control programs such that they elicited an increase in DF incidence. Malaysia reported a strong increase in A. albopictus abundance during lockdown, and India noted an increase in the density of both A. albopictus and A. aegypti, but greater for A. aegypti (Daniel Reegan et al., 2020; Ong et al., 2021). A model by Cavany et al. (Cavany et al., 2021) revealed that initiating lockdown during or after the seasonal peak in infections led to many more infections, while that before the high epidemic period had almost no impact. This demonstrates that mosquito abundance has a robust effect in geographic areas where dengue virus circulation is already established in the mosquito population. That is, lockdown may increase mosquito density, which can exacerbate DF epidemics but not cause them, as the findings demonstrated little correlation between vector indices and human outbreaks (Bowman et al., 2014). On the other hand, local human movements may be a more important driver of dengue virus amplification and spread in view of the restricted movement range of mosquito vectors (Getis et al., 2003; Stoddard et al., 2013). The reduced human movement in cross-regional travel and house-to-house movements may be helpful for limiting the spatial distribution of infections, thereby decreasing the entire level of incidence of the country. Sheng et al. reported that the dengue epidemic was dramatically reduced due to movement restrictions in Yunnan province and was confined to only one city where a dengue epidemic had previously occurred, indicating that restricting intercity movement could not reduce an established outbreak but efficiently blocked city-to-city and urban-to-suburban spread (Sheng et al., 2022).

This suppression of inter-urban transmission is extremely important for both decelerating the spatial spread of dengue virus and reducing the co-circulation of multiple epidemic strains in one location. Furthermore, it may hinder the introduction of a new strain and thereby reduce DF incidence. With the easing of movement restrictions, some factories have reopened and people are allowed to go out on a limited basis, allowing stagnant water to be cleared and mosquito breeding sites to be reduced. Meanwhile, community-wide lockdown measures are carried out dynamically, impeding the geographic extension of dengue virus and increasingly limiting DF-affected places. In this continuous interaction, we presume that despite the varied effect of internal movement restrictions on dengue transmission across countries and over time, the long-term implementation of such measures will be followed by a superimposed effect that is more likely to demonstrate a widely positive effect on reducing dengue transmission at the national level.

Based on our results, we make the following critical recommendations for future DF normalization prevention and control. First, for countries where dengue cases are predominantly imported, aggressive screening and quarantine strategies for international travelers and migrant workers from dengue-endemic areas can achieve good effects. Second, for dengue-endemic countries, identifying dengue latent-infected populations and thorough decontamination of surrounding mosquito vectors, including mosquito eggs, are more important for preventing potential or re-emerging local outbreaks. Additionally, population-wide dengue screening programs should be conducted at specific times to accurately capture DF distribution and to compensate for the drawback of the surveillance system, given that 75% of dengue cases are asymptomatic or unapparent (Roy and Bhattacharjee, 2021). Finally, transnational and cross-regional collaboration, the basis for forming a multinational response and containment strategy and the backbone for achieving complementary healthcare resources, is essential for managing arbovirus epidemics worldwide.

Our study has several limitations. First of all, it is difficult to ascribe changes in a certain disease to a specific NPI, because countries tended to impose comprehensive suites of measures that were highly correlated and varied in time for achieving the strongest and most rapid effect. Therefore, the effectiveness of the NPIs on DF transmission typically occurred as an overall effect, requiring prudent interpretation. Secondly, it is hard to ignore information bias, as the data for dengue cases generated from notification systems are likely more understated than ever. During the COVID-19 pandemic, healthcare-seeking behavior changed drastically due to reasons such as reluctance to attend health facilities for fear of contracting COVID-19, and decreased health utilization (De Filippo et al., 2020). Coupled with the potential misdiagnosis of dengue due to it exhibiting similar initial clinical manifestations to COVID-19, the number of notified DF cases is likely to significantly lower (Roy and Bhattacharjee, 2021). In 2021 in particular, when many countries experienced a COVID-19 onslaught, DF outbreaks may have been severely masked by the terrible burden on the healthcare system (Hossain et al., 2022). Furthermore, with the widespread administration of COVID-19 vaccines, it is worth exploring whether herd immunity to dengue virus has been affected, given that many studies have confirmed serological cross-reactivity between dengue and COVID-19 (Masyeni et al., 2021; Pandey et al., 2021; Santoso et al., 2021). What’s more, the articles included in our review were observational studies, which were limited in obtaining high quality evidence. Therefore, there is a need for further, more rigorous studies to evaluate the efficacy of movement restrictions on dengue transmission by considering various potential influencing factors.

## Conclusion

The multiple NPIs implemented during the COVID-19 pandemic have yielded a large population-based observational study for exploring potential new insights into DF prevention and control strategies. Although the overall effectiveness of NPIs on dengue varied between countries and time due to multiple factors, a significant reduction was observed in most countries. We analyzed the impact of NPIs, including travel restrictions and physical distancing measures, on dengue infection in different countries and time dimensions of dengue incidence. We believe that travel-related interventions are greatly effective for reducing DF imported cases, and internal movement restrictions of constantly varying intensity and range are more likely to mitigate the entire level of dengue transmission by reducing DF spread between regions within the country, which is useful for developing a more comprehensive and sustainable strategy for preventing and controlling DF. Importantly, with the overwhelming occupation of healthcare resources and concerns due to COVID-19, there is an urgent need to focus closely on DF to prevent a global outbreak in the near future.

## Author Contributions

Conceptualization: JL and ZG. Data curation: QW, SD, and XL. Methodology: BY and HH. Formal analysis: QW and SD. Visualization: QW and XL. writing-original draft: QW. writing-review and editing: QW, JL, and ZG. Funding acquisition: JL. All authors contributed to the article and approved the submitted version.

## Funding

This work was funded by the National Key Research and Development Project (2018YFE0208000), the Central Government Guides Local Science and Technology Development Funds to Freely Explore Basic Research Project (2021Szvup171).

## Conflict of Interest

The authors declare that the research was conducted in the absence of any commercial or financial relationships that could be construed as a potential conflict of interest.

## Publisher’s Note

All claims expressed in this article are solely those of the authors and do not necessarily represent those of their affiliated organizations, or those of the publisher, the editors and the reviewers. Any product that may be evaluated in this article, or claim that may be made by its manufacturer, is not guaranteed or endorsed by the publisher.
