# Supplementary material for: Effects of COVID-19 Non-Pharmacological Interventions on Dengue Infection: A Systematic Review and Meta-Analysis
Source: Front Cell Infect Microbiol. 2022 May 19;12:892508. doi: 10.3389/fcimb.2022.892508 (PMC9162155; doi:10.3389/fcimb.2022.892508)

Supplementary Figure 1. A subgroup meta-analysis for the articles with the same study site and analytic methods data but different collection period in non-exposed group. “0”: the study with less than two years of observation in the control group. “1”: the study with more than two years of observation in the control group.

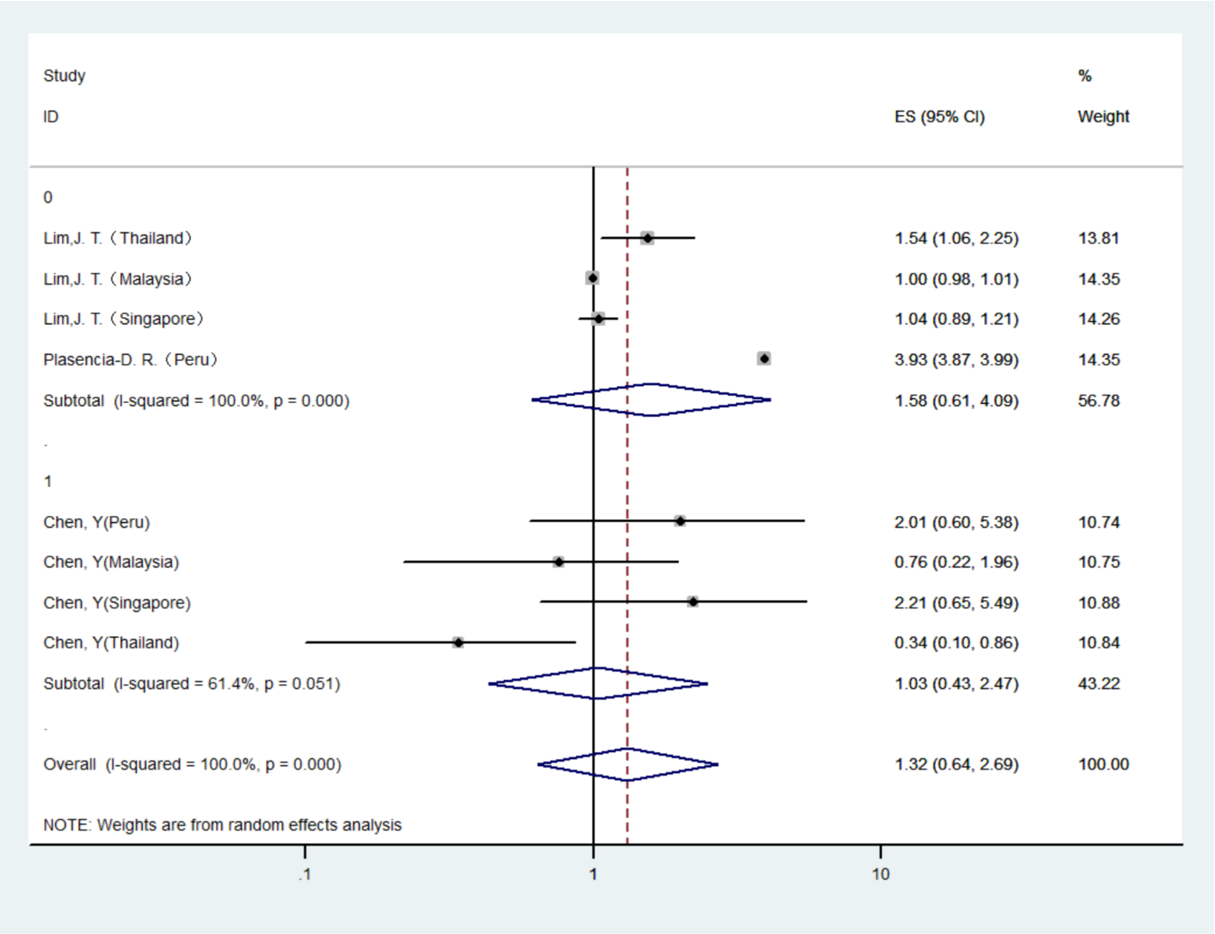

Supplement: Supplementary file 1 [file DataSheet_1.pdf]
