# Supplementary material for: Effects of COVID-19 Non-Pharmacological Interventions on Dengue Infection: A Systematic Review and Meta-Analysis
Source: Front Cell Infect Microbiol. 2022 May 19;12:892508. doi: 10.3389/fcimb.2022.892508 (PMC9162155; doi:10.3389/fcimb.2022.892508)
Supplement: Supplementary file 2 [file DataSheet_2.pdf]

A.

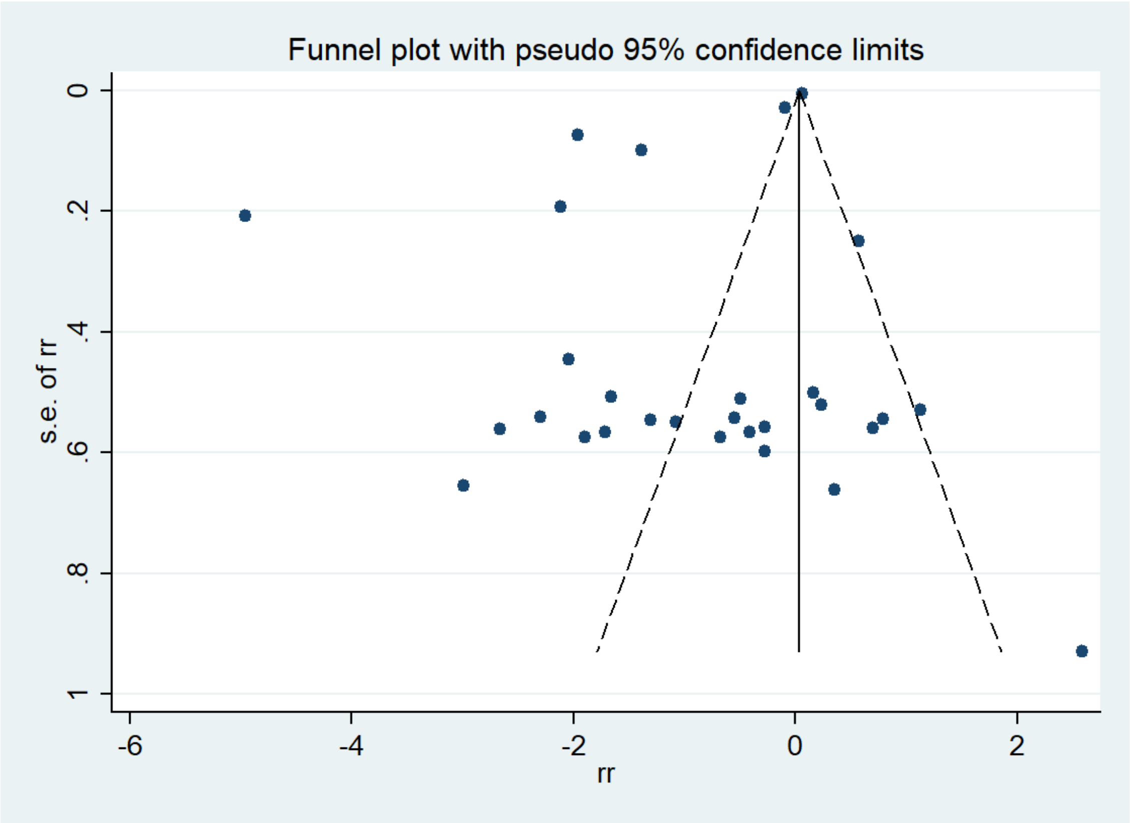

B.

```
Begg's Test

  adj. Kendall's Score (P-Q) =       78
          Std. Dev. of Score =    53.31
           Number of Studies =       29
                           z =     1.46
                    Pr > |z| =    0.143
                           z =     1.44 (continuity corrected)
                    Pr > |z| =    0.149 (continuity corrected)
```

Egger's test

Std_Eff	Coef.	Std. Err.	t	P>|t|	[95% Conf.	Interval]
slope	.0654052	.0360586	1.81	0.081	-.008581	.1393914
bias	-3.923013	1.416651	-2.77	0.010	-6.829742	-1.016285

Supplementary Figure 2. Publication of bias test results. A. Funnel plot. B. Begg's Test and Egger's test.
