# Supplementary material for: Effects of COVID-19 Non-Pharmacological Interventions on Dengue Infection: A Systematic Review and Meta-Analysis
Source: Front Cell Infect Microbiol. 2022 May 19;12:892508. doi: 10.3389/fcimb.2022.892508 (PMC9162155; doi:10.3389/fcimb.2022.892508)
Supplement: Supplementary file 3 [file DataSheet_3.pdf]

A.

Quality assessment							No of patients		Effect		Quality	Importance
No of studies	Design	Risk of bias	Inconsistency	Indirectness	Imprecision	Other considerations			Relative (95% CI)	Absolute		
RR (follow-up median 6 months)												
9	observational studies	serious[1]	serious[1]	no serious indirectness	no serious imprecision	reporting bias strong association[1] increased effect for RR ~1[1]	-	-	RR 0.39 (0.28 to 0.55)	-	⊕OOO VERY LOW	IMPORTANT
								0%		-		

B.

Quality assessment							No of patients		Effect		Quality	Importance
No of studies	Design	Risk of bias	Inconsistency	Indirectness	Imprecision	Other considerations	NPIs		Relative (95% CI)	Absolute		
Relative Risk (follow-up mean 1 years)												
29	observational studies	no serious risk of bias	serious[1]	no serious indirectness	no serious imprecision	strong association[1] increased effect for RR ~1[1]	-	-	RR 0.39 (0.28 to 0.55)	-	⊕⊕⊕O MODERATE	CRITICAL
								0%		-		

[1] No explanation was provided

*The basis for the **assumed risk** (e.g. the median control group risk across studies) is provided in footnotes. The **corresponding risk** (and its 95% confidence interval) is based on the assumed risk in the comparison group and the **relative effect** of the intervention (and its 95% CI).
CI: Confidence interval; **RR:** Risk ratio;

GRADE Working Group grades of evidence
High quality: Further research is very unlikely to change our confidence in the estimate of effect.
Moderate quality: Further research is likely to have an important impact on our confidence in the estimate of effect and may change the estimate.
Low quality: Further research is very likely to have an important impact on our confidence in the estimate of effect and is likely to change the estimate.
Very low quality: We are very uncertain about the estimate.

Supplementary Figure 3. The GRADE assessment for evidence. A and B correspond to different statistical analysis groupings described above.
