# Supplementary material for: Effects of COVID-19 Non-Pharmacological Interventions on Dengue Infection: A Systematic Review and Meta-Analysis
Source: Front Cell Infect Microbiol. 2022 May 19;12:892508. doi: 10.3389/fcimb.2022.892508 (PMC9162155; doi:10.3389/fcimb.2022.892508)
Supplement: Supplementary file 4 [file DataSheet_4.docx]

Search strategy in PubMed

Query Box:

((dengue[Title/Abstract]) OR (DENF[Title/Abstract]) OR (breakbone[Title/Abstract]) OR (break-bone[Title/Abstract])OR (arboviruses[Title/Abstract]) OR (arboviral[Title/Abstract]) OR (arbovirus[Title/Abstract]) OR (mosquito-borne[Title/Abstract]) OR (arthropod- borne[Title/Abstract])) AND ((COVID-19[Title/Abstract]) OR (2019-nCoV[Title/Abstract]) OR (Coronavirus disease-19[Title/Abstract]) OR (2019 novel coronavirus disease[Title/Abstract]) OR (COVID 19[Title/Abstract]) OR (SARS-CoV-2[Title/Abstract]) OR (SARS CoV 2[Title/Abstract])) Filters: from 2019/12/31 - 2022/3/30 Sort by: Publication Date
