# Supplementary material for: Effects of COVID-19 Non-Pharmacological Interventions on Dengue Infection: A Systematic Review and Meta-Analysis
Source: Front Cell Infect Microbiol. 2022 May 19;12:892508. doi: 10.3389/fcimb.2022.892508 (PMC9162155; doi:10.3389/fcimb.2022.892508)
Supplement: Supplementary file 5 [file DataSheet_5.docx]

Meta-regression Number of obs = 9

REML estimate of between-study variance tau2 = .036

% residual variation due to heterogeneity I-squared_res = 00.62%

Proportion of between-study variance explained Adj R-squared = 51.88%

Joint test for all covariates Model F(3.5) = 3.48

With Knapp-Hartung modification Prob > F = 0.1066

| _ES | Coef. | Std. Err | t | p>\| t \| | [95% Conf. Interval] | |
| --- | --- | --- | --- | --- | --- | --- |
| dummy_country~2 | 0.4392591 | 0.1487397 | 2.95 | 0.032 | 0.0569114 | 0.8216067 |
| dummy_ct2 | 0.1668498 | 0.1748998 | 0.95 | 0.384 | -0.2827445 | 0.6164442 |
| dummy_hot2 | -0.0886173 | 0.1692904 | -0.52 | 0.623 | -0.5237922 | 0.3465576 |
| _cons | 0.0649868 | 0.211176 | 0.31 | 0.771 | -0.4778584 | 0.607832 |

‘country’: Study site, a binary variable. The study site is in an area where dengue is endemic, which is identified ‘yes’.

‘ct’: Time of the control group, a binary variable. The value greater than 1 year is identified ‘yes’.

‘hot’: Peak of dengue epidemic season(July-November), a binary variable. The study time included the peak season ,which is identified ‘yes’.
