# Supplementary material for: Effects of COVID-19 Non-Pharmacological Interventions on Dengue Infection: A Systematic Review and Meta-Analysis
Source: Front Cell Infect Microbiol. 2022 May 19;12:892508. doi: 10.3389/fcimb.2022.892508 (PMC9162155; doi:10.3389/fcimb.2022.892508)
Supplement: Supplementary file 6 [file DataSheet_6.docx]

Supplementary Text 3.The extracted information for implementation of NPIs in countries and regions from included studies.

**China(Guangdong, Yunnan)**

**Weeks 4-7(2020)**: Lockdown, travel restrictions, crowd prohibition, and compulsory health quarantine.

**Weeks 9-19(2020)**: Residents from or with a history of travel to overseas countries and territories needed to be quarantined for 14 days and receive health management. International flights were subsequently cut down, and travel was restricted to a specific airline.

**Weeks 20-53(2020)**: Schools returned to a normal routine, but travel-related and case-based NPIs continued to be implemented.

**Taiwan, China**

**January 2020:** Quarantined arrivals.

**March 2020:** Forbidden large-scale public gathering, prohibited travels from foreign nationals.

**April 2020:** Announce social-distancing measures.

**Germany**

**10 March 2020:** Banning mass gatherings started, followed by gradual closing of schools and day care facilities, and a world-wide non-essential travel ban.

**23 March 2020:** Contact with one other person outside of one’s household was allowed. Shops, restaurants, hairdressers and other salons requiring close physical contact were closed; being outside for grocery shopping, medical appointments and sports was permitted. In addition, international travel restrictions and quarantine measures for those entering Germany were put in place for those traveling from areas with high COVID-19 rates.

**20 April 2020:** The first loosening of measures started, by allowing shops of certain sizes to reopen, followed by allowing for larger (though restricted) numbers of people to gather. Different levels of contact restrictions, promotion of hand-hygiene, and the use of face masks in closed (public) spaces such as public transport, stores and the workplace, remained in place till the end of the observational period (week 32-2020).

**Switzerland**

**16 March 2020:** Most non-essential businesses were closed, meeting exceeding 5 persons were prohibited, travel restrictions.

**11 May 2020:** Restaurants opened for parties restricted to 4 persons.

**15 June 2020 :** Lifted for travel to neighboring countries.

**Malaysia**

**18 March to 9 June 2020:** 90% of people were restricted to their homes, and 10% of essential workers were allowed to carry out their daily activities for the whole country.

**Siri Lanka**

**12 March 2020:** Closing all schools since 12th March.

**20 March 2020**: Declaring public holidays and imposing quarantine curfew throughout the entire island ,People were encouraged to stay at home and a working from home policy was introduced. Complete international travel restriction was implemented from 20th March closing airports.

**11 May 2020 :** The government decided to restore normalcy in life while maintaining quarantine curfew.

**28 June 2020:** The curfew was completely lifted.

**Thailand**

**March** **2020**: The reduction of movements through workplace closures and mass gatherings were implemented and heavily enforced in these countries, which have led to a large decrease in time spent in workplaces over a period of 2 to 3 months before gradual relaxation of measures.

**Australia**

**Mid-March 2020:** Travel restrictions, lockdown, emphasis on hygiene.

**Singapore**

**7 April to 1 June 2020：**During this period, around 95% of workplaces were closed, including all schools and recreational facilities, and all construction sites. All migrant construction workers were quarantined in their residences during this period.

**Brazil**

**Weeks 11 (2020):** Included temporary closing of services such as theaters, museums, libraries, state cultural centers, shopping malls, academies, concerts, religious celebrations and other public events, as well as gradual suspension of classes in basic and higher education.

**Week 13(2020):** The quarantine was made official, and it was implemented uniformly for all 645 municipalities of state, with the movement of people limited to activities considered essential as food, supply, health, banks, cleanliness, security and social communication, later observed the permanent use of face masks.

**Week 23(2020):** The government began to regionalize the measures of social distancing.

**Levels of implementation intensity**

The emergency response levels for COVID-19 can be classified as three levels based on the stringency of those measures took by government on public. The first level is strictest, including lockdown, in which public service places, schools and workplaces were closed and citizens were forbidden to gather outside, travel restriction and compulsory health quarantine. During the second level period, some important public places, schools and works were gradually restored normal, and the international flights were cutdown and passengers from those flights must to be quarantined when they disembark. The third level indicated that things was go to normal, but people should take good personal protection in public places and reduce the aggregation, most importantly, the need for mandatory quarantine for entry of persons from abroad was still stressed.
