# Supplementary material for: Effects of COVID-19 Non-Pharmacological Interventions on Dengue Infection: A Systematic Review and Meta-Analysis
Source: Front Cell Infect Microbiol. 2022 May 19;12:892508. doi: 10.3389/fcimb.2022.892508 (PMC9162155; doi:10.3389/fcimb.2022.892508)
Supplement: Supplementary file 7 [file Table_1.docx]

Supplementary Table 1. Quality Assessment of the included studies using modified Newcastle-Ottawa Scale (NOS)

| Study | Selection | | | | Comparability | | Outcome | | | Total  (max score:9*) |
| --- | --- | --- | --- | --- | --- | --- | --- | --- | --- | --- |
|  | *Representativeness of the exposed cohort*  *(max score:*)* | *Selection of non-exposed*  *(max score:*)* | *Ascertainment of exposure*  *(max score:*)* | *Demonstration that outcome of interest was not present at start of study*  *(max score:*)* | *Comparable for onset seasons*  *(max score:*)* | *Study controls for other factors*  *(max score:*)* | *Assessment of outcome*  *(max score:*)* | *Was follow up long enough for outcomes to occur(max score:*)* | *Adequacy of follow up(max score:*)* |  |
| Xiao, J.  [1] | * | * | * | * | * | * | * | * | - | 8* |
| Ullrich, A.  [2] | * | * | * | * | * | * | * | - | - | 7* |
| Steffen, R.  [3] | * | * | * | * | * | - | * | - | - | 6* |
| Lim,J. T  [4] | * | - | * | * | * | * | * | * | - | 7* |
| Rahim, M. H.  [5] | * | - | * | * | - | - | * | - | - | 4* |
| Plasencia-D. R.  [6] | * | * | - | * | * | * | * | * | - | 7* |
| Song-Q.O  [7] | * | * | * | * | * | * | * | * | - | 8* |
| Niriella, M. A.  [8] | * | - | - | * | * | - | * | - | - | 4* |
| Liyanage, P.  [9] | * | * | * | * | * | * | * | - | - | 7* |
| Lim, J. T.  [10] | * | * | * | * | * | * | * | - | - | 7* |
| Lim, J. T.  [11] | * | * | * | * | * | * | * | * | - | 8* |
| Li, N.  [12] | * | * | * | * | * | - | * | * | - | 7* |
| Conceição, G. M. S  [13] | * | - | * | * | - | * | * | * | - | 6* |
| Bright, A  [14] | * | * | * | * | * | - | * | - | - | 6* |
| Lai, C.C.  [15] | * | - | * | * | * | - | * | * | - | 6* |
| Chen, Y.  [16] | * | * | * | * | * | * | * | * | *a | 9* |
| Chavhan, S. S  [17] | * | - | - | - | * | - | * | * | - | 4* |
| Lu, X  [18] | * | * | * | * | * | - | * | * | - | 7* |

Detailed item and criteria in above modified Newcastle-Ottawa Scale (NOS) as following:

1.Selection of non-exposed: the incidence of the non-exposed can represent the history incidence level *

2. Ascertainment of exposure: the article detailed the timing of NPIs for COVID-19*

3. Demonstration that outcome of interest was not present at start of study: the non-exposed group was not exposed to NPIs for COVID-19*

4. Comparable for onset seasons: incidence rates in the exposed and non-exposed groups were compared over the same period*

5. Was follow up long enough for outcomes to occur: the observation time of exposure group included the peak of dengue epidemic*

6. Adequacy of follow up: the influence factors of underreporting were the same in the exposed and non-exposed groups*

a. The study found no evidence that the under-reporting rate was higher or lower than past years.
