# Supplementary material for: Effects of COVID-19 Non-Pharmacological Interventions on Dengue Infection: A Systematic Review and Meta-Analysis
Source: Front Cell Infect Microbiol. 2022 May 19;12:892508. doi: 10.3389/fcimb.2022.892508 (PMC9162155; doi:10.3389/fcimb.2022.892508)
Supplement: Supplementary file 8 [file Table_2.docx]

Supplementary Table 2. A list of the full-text studies excluded and reasons for their exclusion.

| Studies | Exclusion Reason |
| --- | --- |
| Wiyono, L., Rocha, I., Cedeño, T., Miranda, A. V., & Lucero-Prisno Iii, D. E. (2021). Dengue and COVID-19 infections in the ASEAN region: a concurrent outbreak of viral diseases. Epidemiology and health, 43, e2021070. doi.org/10.4178/epih.e2021070 | Review |
| Sarkar, S., Khanna, P., & Singh, A. K. (2021). Impact of COVID-19 in patients with concurrent co-infections: A systematic review and meta-analyses. Journal of medical virology, 93(4), 2385–2395. doi.org/10.1002/jmv.26740 | Review |
| Vicente, C. R., Silva, T., Pereira, L. D., & Miranda, A. E. (2021). Impact of concurrent epidemics of dengue, chikungunya, zika, and COVID-19. Revista da Sociedade Brasileira de Medicina Tropical, 54, e08372020. doi.org/10.1590/0037-8682-0837-2020 | No comparison |
| Stringari, L. L., de Souza, M. N., de Medeiros Junior, N. F., Goulart, J. P., Giuberti, C., Dietze, R., et al.(2021). Covert cases of Severe Acute Respiratory Syndrome Coronavirus 2: An obscure but present danger in regions endemic for Dengue and Chikungunya viruses. PloS one, 16(1), e0244937. doi.org/10.1371/journal.pone.0244937 | Wrong study design |
| Saavedra-Velasco, M., Chiara-Chilet, C., Pichardo-Rodriguez, R., Grandez-Urbina, A., & Inga-Berrospi, F. (2020). Coinfección entre dengue y COVID-19: Necesidad de abordaje en zonas endémicas [Coinfection between dengue and covid-19: need for approach in endemic zones.]. Revista de la Facultad de Ciencias Medicas (Cordoba, Argentina), 77(1), 52–54. doi.org/10.31053/1853.0605.v77.n1.28031 | Review |
| Silva, S., Magalhães, J., & Pena, L. (2020). Simultaneous Circulation of DENV, CHIKV, ZIKV and SARS-CoV-2 in Brazil: an Inconvenient Truth. One health (Amsterdam, Netherlands), 12, 100205. doi.org/10.1016/j.onehlt.2020.100205 | No detailed data |
| Semenza, J. C., & Paz, S. (2021). Climate change and infectious disease in Europe: Impact, projection and adaptation. The Lancet regional health. Europe, 9, 100230. doi.org/10.1016/j.lanepe.2021.100230 | Review |
| Ribeiro, V., Telles, J. P., & Tuon, F. F. (2020). Arboviral diseases and COVID-19 in Brazil: Concerns regarding climatic, sanitation, and endemic scenario. Journal of medical virology, 92(11), 2390–2391. doi.org/10.1002/jmv.26079 | Letter to the editor |
| Rahman, M. M., Bodrud-Doza, M., Shammi, M., Md Towfiqul Islam, A. R., & Moniruzzaman Khan, A. S. (2021). COVID-19 pandemic, dengue epidemic, and climate change vulnerability in Bangladesh: Scenario assessment for strategic management and policy implications. Environmental research, 192, 110303. doi.org/10.1016/j.envres.2020.110303 | Wrong study design |
| Poveda G. (2020). Concomitant malaria, dengue and COVID-19: an extraordinary challenge for Colombia's public health system. Current opinion in environmental sustainability, 46, 23–26. doi.org/10.1016/j.cosust.2020.10.006 | Review |
| Pandey, K., , Dumre, S. P., , Dhimal, M., , Pun, S. B., , Shah, Y., , Fernandez, S., , Morita, K., , & Pandey, B. D., (2021). The Double Burden of COVID-19 and Dengue in Nepal: The challenges ahead. Kathmandu University medical journal (KUMJ), 19(73), 140–142. | No detailed data |
| Núñez-López, M., Alarcón Ramos, L., & Velasco-Hernández, J. X. (2021). Migration rate estimation in an epidemic network. Applied mathematical modelling, 89, 1949–1964. doi.org/10.1016/j.apm.2020.08.025 | Wrong study design |
| Nicolete, V. C., Rodrigues, P. T., Johansen, I. C., Corder, R. M., Tonini, J., Cardoso, M. A., et al. (2021). Interacting Epidemics in Amazonian Brazil: Prior Dengue Infection Associated With Increased Coronavirus Disease 2019 (COVID-19) Risk in a Population-Based Cohort Study. Clinical infectious diseases : an official publication of the Infectious Diseases Society of America, 73(11), 2045–2054. doi.org/10.1093/cid/ciab410 | Wrong study design |
| Nakandakari Gomez M, Marín-Macedo H, Seminario-Vilca R.(2021). Dengue with signs of alarm and Leptospirosis in a pediatric patient with COVID-19. Revista de la Facultad de Medicina Humana, 21(2): 24. | Wrong research objectives |
| Muñoz, Á. G., Chourio, X., Rivière-Cinnamond, A., Diuk-Wasser, M. A., Kache, P. A., Mordecai, E. A., et al. (2020). AeDES: a next-generation monitoring and forecasting system for environmental suitability of Aedes-borne disease transmission. Scientific reports, 10(1), 12640. doi.org/10.1038/s41598-020-69625-4 | Wrong research objectives |
| Morgan, J., Strode, C., & Salcedo-Sora, J. E. (2021). Climatic and socio-economic factors supporting the co-circulation of dengue, Zika and chikungunya in three different ecosystems in Colombia. PLoS neglected tropical diseases, 15(3), e0009259. doi.org/10.1371/journal.pntd.0009259 | Wrong study design |
| Lokossou, V. K., Bunyoga, D., Sombie, I., & Okolo, S. (2021). Coexistence and management of COVID-19 pandemic with other epidemics in West Africa: lessons learnt and policy implications. The Pan African medical journal, 38, 341. doi.org/10.11604/pamj.2021.38.341.27901 | Review |
| Lin, S. F., Lai, C. C., Chao, C. M., & Tang, H. J. (2021). Impact of COVID-19 preventative measures on dengue infections in Taiwan. Journal of medical virology, 93(7), 4063–4064. doi.org/10.1002/jmv.26650 | Letter to the editor |
| Junaid Tahir, M., Rizwan Siddiqi, A., Ullah, I., Ahmed, A., Dujaili, J., & Saqlain, M. (2020). Devastating urban flooding and dengue outbreak during the COVID-19 pandemic in Pakistan. Medical journal of the Islamic Republic of Iran, 34, 169. doi.org/10.47176/mjiri.34.169 | Letter to the editor |
| Jiang, L., Liu, Y., Su, W., Liu, W., & Yang, Z. (2021). Decreased dengue cases attributable to the effect of COVID-19 in Guangzhou in 2020. PLoS neglected tropical diseases, 15(5), e0009441. doi.org/10.1371/journal.pntd.0009441 | Wrong study design |
| Haqqi, A., Awan, U. A., Ali, M., Saqib, M., Ahmed, H., & Afzal, M. S. (2021). COVID-19 and dengue virus coepidemics in Pakistan: A dangerous combination for an overburdened healthcare system. Journal of medical virology, 93(1), 80–82. doi.org/10.1002/jmv.26144 | Letter to the editor |
| Dantés, H. G., Manrique-Saide, P., Vazquez-Prokopec, G., Morales, F. C., Siqueira Junior, J. B., Pimenta, F., et al. (2020). Prevention and control of Aedes transmitted infections in the post-pandemic scenario of COVID-19: challenges and opportunities for the region of the Americas. Memorias do Instituto Oswaldo Cruz, 115, e200284. doi.org/10.1590/0074-02760200284 | Review |
| Daniel Reegan, A., Rajiv Gandhi, M., Cruz Asharaja, A., Devi, C., & Shanthakumar, S. P. (2020). COVID-19 lockdown: impact assessment on Aedes larval indices, breeding habitats, effects on vector control programme and prevention of dengue outbreaks. Heliyon, 6(10), e05181. doi.org/10.1016/j.heliyon.2020.e05181 | Wrong research outcome (Aedes larval indices) |
| Cheng, X., Hu, J., Luo, L., Zhao, Z., Zhang, N., Hannah, M. N., et al. (2021). Impact of interventions on the incidence of natural focal diseases during the outbreak of COVID-19 in Jiangsu Province, China. Parasites & vectors, 14(1), 483. doi.org/10.1186/s13071-021-04986-x | No detailed data |
| Cavany, S. M., España, G., Vazquez-Prokopec, G. M., Scott, T. W., & Perkins, T. A. (2021). Pandemic-associated mobility restrictions could cause increases in dengue virus transmission. PLoS neglected tropical diseases, 15(8), e0009603. doi.org/10.1371/journal.pntd.0009603 | Wrong research outcome (DENV transmission) |
| Castañeda-Gómez, J., González-Acosta, C., Jaime-Rodríguez, J. L., Villegas-Trejo, A., & Moreno-García, M. (2021). COVID-19 and its impact on the control of Aedes (Stegomyia) aegypti mosquito and epidemiological surveillance of arbovirus infections. Gaceta medica de Mexico, 157(2), 187–193. doi.org/10.24875/GMM.M21000546 | Review |
| Brady, O., & Wilder-Smith, A. (2021). What Is the Impact of Lockdowns on Dengue?. Current infectious disease reports, 23(2), 2. doi.org/10.1007/s11908-020-00744-9 | Review |
| Bardhan, M., Pramanik, D., Riyaz, R., Hasan, M. M., & Essar, M. Y. (2021). Dual burden of Zika and COVID-19 in India: challenges, opportunities and recommendations. Tropical medicine and health, 49(1), 83. doi.org/10.1186/s41182-021-00378-0 | Letter to the editor |
| Asawapaithulsert, P., Pisutsan, P., & Matsee, W. (2021). Coincidence of fever following COVID-19 vaccine and endemic tropical diseases: a challenge to clinicians during the global rollout of COVID-19 vaccination. Journal of travel medicine, 28(6), taab109. doi.org/10.1093/jtm/taab109 | Letter to the editor |
| Adegbija, O., Walker, J., Smoll, N., Khan, A., Graham, J., & Khandaker, G. (2021). Notifiable diseases after implementation of COVID-19 public health prevention measures in Central Queensland, Australia. Communicable diseases intelligence (2018), 45, 10.33321/cdi.2021.45.11. doi.org/10.33321/cdi.2021.45.11 | No detailed data |
| Aborode, A. T., Corriero, A. C., Fajemisin, E. A., Hasan, M. M., Kazmi, S. K., & Olajiga, O. (2021). Dengue and Coronavirus disease (COVID-19) syndemic: Double threat to an overburdened healthcare system in Africa. The International journal of health planning and management, 10.1002/hpm.3334. Advance online publication. doi.org/10.1002/hpm.3334 | Letter to the editor |
| Roster, K., Connaughton, C., & Rodrigues, F. A. (2021). 1222 Estimating the causal effect of mobility on Dengue during the COVID-19 pandemic. International Journal of Epidemiology, 50(Supplement_1), dyab168-573. | No detailed data |
| Jansen, C. C., Darbro, J. M., Birrell, F. A., Shivas, M. A., & van den Hurk, A. F. (2021). Impact of COVID-19 Mitigation Measures on Mosquito-Borne Diseases in 2020 in Queensland, Australia. Viruses, 13(6), 1150. doi.org/10.3390/v13061150 | Study site and analytic method duplication |
| Webb C. E. (2020). Reflections on a highly unusual summer: bushfires, COVID-19 and mosquito-borne disease in NSW, Australia. Public health research & practice, 30(4), 3042027. 10.17061/phrp3042027 | Review |
| Uwishema, O., Nnagha, E. M., Chalhoub, E., Nchasi, G., Mwazighe, R. M., Akin, B. T., Adanur, I., & Onyeaka, H. (2021). Dengue fever outbreak in Cook Island: A rising concern, efforts, challenges, and future recommendations. Journal of medical virology, 93(11), 6073–6076. doi.org/10.1002/jmv.27223 | Review |
| Silvestre, O. M., Costa, L. R., Lopes, B., Barbosa, M. R., Botelho, K., Albuquerque, K., Souza, A., Coelho, L. A., de Oliveira, A. J., Barantini, C. B., Neves, S., Nadruz, W., Maguire, J. H., & Fernandes-Silva, M. M. (2021). Previous Dengue Infection and Mortality in Coronavirus Disease 2019 (COVID-19). Clinical infectious diseases : an official publication of the Infectious Diseases Society of America, 73(5), e1219–e1221. doi.org/10.1093/cid/ciaa1895 | Wrong research objectives |
| Seposo X. T. (2021). Dengue at the time of COVID-19 in the Philippines. Western Pacific surveillance and response journal : WPSAR, 12(2), 38–39. doi.org/10.5365/wpsar.2020.11.2.015 | Review |
| Sasmono, R. T., & Santoso, M. S. (2022). Movement dynamics: reduced dengue cases during the COVID-19 pandemic. The Lancet. Infectious diseases, S1473-3099(22)00062-7. Advance online publication. doi.org/10.1016/S1473-3099(22)00062-7 | Review |
| Rahman, F. I., Ether, S. A., & Islam, M. R. (2022). Upsurge of Dengue Prevalence During the Third Wave of COVID-19 Pandemic in Bangladesh: Pouring Gasoline to Fire. Clinical pathology (Thousand Oaks, Ventura County, Calif.), 15, 2632010X221076068. doi.org/10.1177/2632010X221076068 | Review |
| Prasertbun, R., Mori, H., Mahittikorn, A., Siri, S., & Naito, T. (2022). Pneumonia, influenza, and dengue cases decreased after the COVID-19 pandemic in Thailand. Tropical medicine and health, 50(1), 27. doi.org/10.1186/s41182-022-00419-2 | No detailed data |
| Phadke, R., Mohan, A., Çavdaroğlu, S., Dapke, K., Costa, A., Riaz, M., Hashim, H. T., Essar, M. Y., & Ahmad, S. (2021). Dengue amidst COVID-19 in India: The mystery of plummeting cases. Journal of medical virology, 93(7), 4120–4121. doi.org/10.1002/jmv.26987 | Letter to the editor |
| Surendran, S. N., Nagulan, R., Sivabalakrishnan, K., Arthiyan, S., Tharsan, A., Jayadas, T., Raveendran, S., Kumanan, T., & Ramasamy, R. (2022). Reduced dengue incidence during the COVID-19 movement restrictions in Sri Lanka from March 2020 to April 2021. BMC public health, 22(1), 388. doi.org/10.1186/s12889-022-12726-8 | Study site and analytic method duplication |
| Olive, M. M., Baldet, T., Devillers, J., Fite, J., Paty, M. C., Paupy, C., Quénel, P., Quillery, E., Raude, J., Stahl, J. P., Thiann-Bo-Morel, M., & Roiz, D. (2020). The COVID-19 pandemic should not jeopardize dengue control. PLoS neglected tropical diseases, 14(9), e0008716. doi.org/10.1371/journal.pntd.0008716 | Review |
| Nicolete, V. C., Rodrigues, P. T., Johansen, I. C., Corder, R. M., Tonini, J., Cardoso, M. A., de Jesus, J. G., Claro, I. M., Faria, N. R., Sabino, E. C., Castro, M. C., & Ferreira, M. U. (2021). Interacting Epidemics in Amazonian Brazil: Prior Dengue Infection Associated With Increased Coronavirus Disease 2019 (COVID-19) Risk in a Population-Based Cohort Study. Clinical infectious diseases : an official publication of the Infectious Diseases Society of America, 73(11), 2045–2054. doi.org/10.1093/cid/ciab410 | Wrong research objectives |
| Navarro, J. C., Arrivillaga-Henríquez, J., Salazar-Loor, J., & Rodriguez-Morales, A. J. (2020). COVID-19 and dengue, co-epidemics in Ecuador and other countries in Latin America: Pushing strained health care systems over the edge. Travel medicine and infectious disease, 37, 101656. doi.org/10.1016/j.tmaid.2020.101656 | Letter to the editor |
| Moise, I. K., Ortiz-Whittingham, L. R., Omachonu, V., Clark, M., & Xue, R. D. (2021). Fighting mosquito bite during a crisis: capabilities of Florida mosquito control districts during the COVID-19 pandemic. BMC public health, 21(1), 687. doi.org/10.1186/s12889-021-10724-w | Wrong research objectives |
| Mohan, A., Fakhor, H., Nimavat, N., Wara, U. U., Lal, P. M., Costa, A., Ahmad, S., & Essar, M. Y. (2021). Dengue and COVID-19: A risk of coepidemic in Ethiopia. Journal of medical virology, 93(10), 5680–5681. doi.org/10.1002/jmv.27116 | Letter to the editor |
| Lamsal M. (2021). The Risk of Dengue and Scrub Typhus Overshadowed by COVID-19 in Nepal. Asia-Pacific journal of public health, 33(5), 679–680. doi.org/10.1177/10105395211012907 | Review |
| Khatri, G., Hasan, M. M., Shaikh, S., Mir, S. L., Sahito, A. M., Priya, Rocha, I., & Elmahi, O. (2022). The simultaneous crises of dengue and COVID-19 in Pakistan: a double hazard for the country's debilitated healthcare system. Tropical medicine and health, 50(1), 18. doi.org/10.1186/s41182-022-00410-x | Review |
| Khan, S. A., Webb, C. E., & Abu Kassim, N. F. (2021). Prioritizing mosquito-borne diseases during and after the COVID-19 pandemic. Western Pacific surveillance and response journal : WPSAR, 12(2), 40–41. doi.org/10.5365/wpsar.2020.11.3.017 | Review |
| Islam, Z., Mohanan, P., Bilal, W., Hashmi, T., Rahmat, Z., Abdi, I., Riaz, M., & Essar, M. Y. (2022). Dengue Virus Cases Surge Amidst COVID-19 in Pakistan: Challenges, Efforts and Recommendations. Infection and drug resistance, 15, 367–371. doi.org/10.2147/IDR.S347571 | Review |
| Hossain, M. J., Soma, M. A., Islam, M. R., & Emran, T. B. (2021). Urgent call for actionable measures to fight the current co-epidemic of dengue burden during the SARS-CoV-2 delta variant era in South-Asia. Ethics, medicine, and public health, 19, 100726. doi.org/10.1016/j.jemep.2021.100726 | Letter to the editor |
| Hasan, M. M., Sahito, A. M., Muzzamil, M., Mohanan, P., Islam, Z., Billah, M. M., Islam, M. J., & Essar, M. Y. (2022). Devastating dengue outbreak amidst COVID-19 pandemic in Bangladesh: an alarming situation. Tropical medicine and health, 50(1), 11. doi.org/10.1186/s41182-022-00401-y | Review |
| Harapan, H., Ryan, M., Yohan, B., Abidin, R. S., Nainu, F., Rakib, A., Jahan, I., Emran, T. B., Ullah, I., Panta, K., Dhama, K., & Sasmono, R. T. (2021). Covid-19 and dengue: Double punches for dengue-endemic countries in Asia. Reviews in medical virology, 31(2), e2161. doi.org/10.1002/rmv.2161 | Review |
| Guo, X., Ma, C., Wang, L., Zhao, N., Liu, S., & Xu, W. (2022). The impact of COVID-19 continuous containment and mitigation strategy on the epidemic of vector-borne diseases in China. Parasites & vectors, 15(1), 78. https://doi.org/10.1186/s13071-022-05187-w | Study site and analytic method duplication |
| Fauziyah, S., Putri, S., Salma, Z., Wardhani, H. R., Hakim, F., Sucipto, T. H., Aquaresta, F., & Soegijanto, S. (2021). How should Indonesia consider its neglected tropical diseases in the COVID-19 era? Hopes and challenges (Review). Biomedical reports, 14(6), 53. doi.org/10.3892/br.2021.1429 | Review |
| Cruz-Lopez, F., Garza-González, E., Morfin-Otero, R., Villarreal-Treviño, L., Rodriguez-Noriega, E., & Martínez-Meléndez, A. (2022). Analysis of influenza and dengue cases in Mexico before and during the COVID-19 pandemic. Infectious diseases (London, England), 54(3), 232–234. doi.org/10.1080/23744235.2021.1999496 | Study site and analytic method duplication |
| Concha-Velasco, F., & Curioso, W. H. (2021). COVID-19 y la necesidad urgente de controlar brotes de dengue y otros arbovirus [COVID-19 and the urgent need to control outbreaks of dengue and other arboviruses]. Revista chilena de infectologia : organo oficial de la Sociedad Chilena de Infectologia, 38(3), 463–464. doi.org/10.4067/S0716-10182021000300463 | Review |
| Butt, M. H., Safdar, A., Amir, A., Zaman, M., Ahmad, A., Saleem, R. T., Misbah, S., Khan, Y. H., & Mallhi, T. H. (2021). Arboviral diseases and COVID-19 coincidence: Challenges for Pakistan's derelict healthcare system. Journal of medical virology, 93(12), 6465–6467. doi.org/10.1002/jmv.27241 | Letter to the editor |
| Berberian G. (2021). Dengue en los inicios de la pandemia de COVID-19 en la Argentina [Dengue at the beginning of the COVID-19 pandemic in Argentina]. Archivos argentinos de pediatria, 119(2), 131–138. doi.org/10.5546/aap.2021.131 | No detailed data |
| Tsheten, T., Wangchuk, S., Wangmo, D., Clements, A., Gray, D. J., & Wangdi, K. (2021). COVID-19 Response and Lessons Learned on Dengue Control in Bhutan. Journal of medical entomology, 58(2), 502–504. doi.org/10.1093/jme/tjaa225 | Letter to the editor |
| Castañeda-Gómez, J., González-Acosta, C., Jaime-Rodríguez, J. L., Villegas-Trejo, A., & Moreno-García, M. (2021). COVID-19 and its impact on the control of Aedes (Stegomyia) aegypti mosquito and epidemiological surveillance of arbovirus infections. COVID-19 y su impacto en el control del mosquito Aedes (Stegomyia) aegypti y la vigilancia epidemiológica de infecciones por arbovirus. Gaceta medica de Mexico, 157(2), 187–193. doi.org/10.24875/GMM.M21000546 | Review |
| Cardona-Ospina, J. A., Arteaga-Livias, K., Villamil-Gómez, W. E., Pérez-Díaz, C. E., Katterine Bonilla-Aldana, D., Mondragon-Cardona, Á., Solarte-Portilla, M., Martinez, E., Millan-Oñate, J., López-Medina, E., López, P., Navarro, J. C., Perez-Garcia, L., Mogollon-Rodriguez, E., Rodríguez-Morales, A. J., & Paniz-Mondolfi, A. (2021). Dengue and COVID-19, overlapping epidemics? An analysis from Colombia. Journal of medical virology, 93(1), 522–527. doi.org/10.1002/jmv.26194 | No detailed data |
| Chavhan, S. S., Kashyap, V., Gokhale, C. N., Adsul, B. B., Gomare, M., Kumbhar, M., Kadam, N., Dhikale, P. T., & Kinge, K. V. (2021). Epidemiological study to assess the impact of COVID-19 pandemic on the occurrence of monsoon-related diseases in the city of Mumbai. Journal of family medicine and primary care, 10(10), 3595–3599. doi.org/10.4103/jfmpc.jfmpc_151_21 | No detailed data |
