# Supplementary material for: Effects of COVID-19 Non-Pharmacological Interventions on Dengue Infection: A Systematic Review and Meta-Analysis
Source: Front Cell Infect Microbiol. 2022 May 19;12:892508. doi: 10.3389/fcimb.2022.892508 (PMC9162155; doi:10.3389/fcimb.2022.892508)
Supplement: Supplementary file 9 [file Table_3.docx]

Supplementary Table 3. Characteristics of studies included in the systematic review and meta-analysis..

| First author | Study site | Endemic or imported | Data source | Data collection period | | Number of cases /incidence（per million） | | Population type or size/million | Analyti-cal model ( Category classification*) | Study factor-s | confounding factor under control | Effect  indicators | Result | stratified analysis |
| --- | --- | --- | --- | --- | --- | --- | --- | --- | --- | --- | --- | --- | --- | --- |
|  |  |  |  | Control group | Exposed group | Control group | Exposed group |  |  |  |  |  |  |  |
| Xiao, J.  [1] | Guangdong, China | NO | NIDSS | Weeks 4-53 in 2015-2019 | Weeks 4-53 in 2020 | 2643 | 50 | GP | BSTS  (B) | NPIs | Seasonality, Time trends | Relative reduction(%)^1^ | 99.3%(99.1%-99.6%)  RR:0.007(0.004,0.009) | YES |
| Ullrich, A.  [2] | Germany | NO | PKA | Weeks 10-32in 2016-2019 | Weeks  10-32 in 2020 | - | - | GP | NBR,TS(C) | NPIs | Seasonality, Time trends | Relative change(Δ%)^2^ | 75.1%(79.5%-  69.9%)  RR:0.249(0.205,0.301) | YES(no detailed data) |
| Steffen,R.  [3] | Switzerland | NO | SFOPH | Weeks 15-26 in2016-2019 | Weeks 15-26 in 2020 | 38 | 4 | GP/8.6369 | -  (A) | Lockdown | - | Reduction(%) | 89.5% | NO |
| Lim, J. T  [4] | Thailand | YES | NDSRS | 2019 | 2020 | - | - | GP | RDD  (C) | SD | Climate, Seasonality | coefficient | 0.43(0.059-0.81）  OR:1.537(1.061,2.247) | NO |
|  | Malaysia | YES | MAMPU | 2019 | 2020 | - | - | GP |  |  |  | coefficient | -0.004(-0.018,-0.012)  OR:0.996 (0.982,1.012) | NO |
|  | Singapore | YES | WIDB | 2019 | 2020 | - | - | GP |  |  |  | coefficient | 0.036(-0.115,0.187)  OR:1.037(0.891,1.206) | NO |
| Rahim, M. H.  [5] | Peninsular Malaysia | YES | CPRC, DOCW | Weeks 10-11 in 2020 | Weeks 12-13, 14-15, 16-17 in 2020 | 7268 | MCOI：4662  MCOⅡ:3075  MCOⅢ: 3505 | GP/32.73 | -  (A) | NPIs | - | Reduction(%) | MCOI:35.88%; MCOⅡ:57.69%  MCOⅢ:51.7%  48.85% per week | NO |
| Plasencia-D. R.  [6] | Peru | YES | CDC | Whole weeks in 2018-2019 | Whole weeks in 2020 | 8.22 | 32.6 | GP | PAF  ,PR  (C) | NPIs | Temperature, Humidity, Autocorrelation | IRR | 3.93(3.87-3.99) | NO |
| Song-Q.O  [7] | Malaysia | YES | DSS | 1^st^ week of 2015 to 52^nd^ week of 2019 | 1^st^ week to 35^th^ week of 2020 | - | - | GP | SARIMA  (B) | Lockdown | Seasonality | The trend of incidence | 1.decrese in the early of lockdown, and then significantly increase in dengue transmission.  2.The abundance of Ae. Albopictus demonstrated a strong linear increment throughout the eight stages of lockdown. | - |
| Niriella, M. A.  [8] | Sri Lanka | YES | EUMH | 1 April to 30 June 2019 | 1 April to 30 June 2020 | 13249 | 3492 | 2.2 | -  (A） | NPIs | - | Reduction(%) | 73.6% | NO |
| Liyanage, P.  [9] | Sri Lanka. | YES | EUMH | January to March in 2015-2020 | April to June in 2020 | - | - | GP | ITS,PR  (A) | Lockdown |  | RR | 0.12(0.08-0.17) | NO |
| Lim, J. T.  [10] | Singapore | YES | WIDB | 1 January 2013 to 1 April in 2020 | April to June in 2020 | - | - | MW | DID  (C） | Quarantine | Weather, Age | Reduction(%) | 68.5% | NO |
|  |  |  |  |  |  |  |  | GW |  | SD |  |  | -63.5% |  |
| Lim, J. T.  [11] | Singapore | YES | WIDB | 2003-2020 | | - | - | GW | DID  (C) | SD | Weather, Age, Time trend | Reduction(%) | -37.2%(19.9%-49.8%）  OR:1.372(1.199,1.498) | NO |
| Li, N.  [12] | Yunnan, China | NO | CDC | 2013-2019 | 2020 | 15000 | 260 | GP/4.7222 | GLM  (C) | Border restrictions | Climate factors, Time trend,  Population size | RR | 0.052 | NO |
|  | Thailand | YES | WPRO IRIS | 2013-2019 | 2020 | 68739 | 50042 | GP/6.98 | -  (A) | NPIs | - | Reduction(%) | 27.2% | NO |
|  | Viet Nam | YES |  | 2013-2019 | 2020 | 137328 | 121398 | GP/9.73386 | -  (A) |  | - | Reduction(%) | 11.6% |  |
|  | Laos | YES |  | 2013-2019 | 2020 | 16712 | 7554 | GP/0.7231211 | -  (A) |  | - | Reduction(%) | 54.8% |  |
|  | Yunnan | NO | CDC | 2013-2019 | 2020 | 2241 | 260 | GP/4.7222 | -  (A) |  | - |  | 88.4% |  |
| Conceição, G. M. S  [13] | Sao Paulo, Brazil | YES | ESD | 1 January to February 6, 2020 | February 6 to August 11, 2020 | - | - | GP | RMNBR  (C) | SD | Seasonality,  Autocorrelation | Reduction(%) | 9.1%(14.2%-3.7%)  RR:0.909(0.858,0.962) | NO |
| Bright, A  [14] | Australia | NO | NNDSS | Mean of January to June 2015-2019 | January to June 2020 | 918 | 192 | GP/25.7 | -  (A) | NPIs | - | Reduction (%) | 79% | NO |
| Lai, C.C.  [15] | Taiwan, China | NO | CDC | January and September in 2019 | January and September in 2020 | 408 | 59 | GP/2.3561 | -  (A) | NPIs | - | Reduction(%) | 85.5% | NO |
| Chen, Y.  [16] | Belize | YES | WHO | April to December 2014-2019 | April to December 2020 | - | - | GP | BRM  (B) | NPIs | Climate variable, Population size,  Time trends | Relative  reduction(%) | -77%  (-94%, 27%)  RR:1.77(0.73, 1.94) | NO |
|  | Bolivia | YES |  |  |  |  |  |  |  |  |  |  | -42%  (-329%, 68%)  RR:1.42(0.32,4.29) | NO |
|  | Brazil | YES |  |  |  |  |  |  |  |  |  |  | -1225%  (-4154%, -11%)  RR:13.25(1.11,42.54) | NO |
|  | Colombia | YES |  |  |  |  |  |  |  |  |  |  | 39%  (-48%, 80%)  RR:0.61(0.2,1.48) | NO |
|  | Costa Rica | YES |  |  |  |  |  |  |  |  |  |  | -26%  (-208%, 60%)  RR:1.26(0.40,3.08) | NO |
|  | Dominican Republic | YES |  |  |  |  |  |  |  |  |  |  | 93%  (82%, 98%)  RR:0.07(0.02,0.18) | NO |
|  | Ecuador | YES |  |  |  |  |  |  |  |  |  |  | 49%  (-33%, 86%)  RR:0.51(0.14,1.33) | NO |
|  | EL Salvador | YES |  |  |  |  |  |  |  |  |  |  | 73%  (32%,92%)  RR:0.27(0.08,0.68) | NO |
|  | Guatemala | YES |  |  |  |  |  |  |  |  |  |  | 87%  (67%, 96%)  RR:0.13(0.04,0.23) | NO |
|  | Honduras | YES |  |  |  |  |  |  |  |  |  |  | -18%  (-177%, 61%)  RR:1.18(0.39,2.77) | NO |
|  | Jamaica | YES |  |  |  |  |  |  |  |  |  |  | 95%  (87%, 99%)  RR:0.05(0.01,0.13) | NO |
|  | Mexico | YES |  |  |  |  |  |  |  |  |  |  | 24%  (-109%,80%)  RR:0.76(0.2,2.09) | NO |
|  | Nicaragua | YES |  |  |  |  |  |  |  |  |  |  | -208%  (-670%,3%)  RR:3.08(0.97,7.70 | NO |
|  | Panama | YES |  |  |  |  |  |  |  |  |  |  | 81%  (56%, 94%)  RR:0.19(0.06,0.44) | NO |
|  | Peru | YES |  |  |  |  |  |  |  |  |  |  | -101%  (-438%, 40%)  RR:2.01(0.60,5.38) | NO |
|  | Venezuela | YES |  |  |  |  |  |  |  |  |  |  | 90%  (75%, 97%)  RR:0.10(0.03,0.25) | NO |
|  | Cambodia | YES |  |  |  |  |  |  |  |  |  |  | 82%  (54%, 95%)  RR:0.18(0.05,0.46) | NO |
|  | Laos | YES |  |  |  |  |  |  |  |  |  |  | 42%  (-43%, 83%)  RR:0.58(0.17,1.43) | NO |
|  | Malaysia | YES |  |  |  |  |  |  |  |  |  |  | 24%  (-96%,78%)  RR:0.76(0.22,1.96) | NO |
|  | Philippines | YES |  |  |  |  |  |  |  |  |  |  | 85%  (62%, 96%)  RR:0.15(0.04,0.38) | NO |
|  | Singapore | YES |  |  |  |  |  |  |  |  |  |  | -121%  (-449%, 35%)  RR:2.21(0.65,5.49) | NO |
|  | Thailand | YES |  |  |  |  |  |  |  |  |  |  | 66%  (14%, 90%)  RR:0.34(0.10,0.86) | NO |
|  | Vietnam | YES |  |  |  |  |  |  |  |  |  |  | 34%  (-66%, 82%)  RR:0.66(0.18,1.66) | NO |
| Lu, X  [17] | Australia | NO | HDE | 2015-2019 | 2020 | - | - | GP | -  (A) | NPIs | - | Reduction(%) | 0.14(0.12, 0.16) | NO |
|  | Vietnam | YES |  |  |  |  |  |  |  |  |  |  | 0.77(0.77,0.78) | NO |
|  | Thailand | YES |  |  |  |  |  |  |  |  |  |  | 0.84(0.83,0.85) | NO |
|  | Indonesia | YES |  |  |  |  |  |  |  |  |  |  | 1.06.(1.05,1.07) | NO |

“*”: (A) One is similar to single-arm design, which adopted the historical control, but all the data including outcomes in exposure group was based on historical data. And the incidence in control and exposure group was directly compared to get the effect value without any confounders controlled. (B) The second way was a comparison between actual incidence and expected incidence of DF during the COVID-19 pandemic to control the influence of time trend on incidence. The expected value usually obtained by using a time series model based on historical onset data of DF before the COVID-19 pandemic. (C) The last approach applied a regression model incorporating various confounding factors to acquire the risk ratio of NPIs.

“1”: Relative reduction(%)=100% × (number of expected cases –number of observed cases)/number of expected cases.

“2”: relative change(Δ%) =100% × ( number of observed cases-number of expected cases)/number of expected cases.

“3”: Adjusted IRR =incidence rate(during COVID-19)/incidence rate（pre-COVID-19）, adjusted by temperature and humidity factors.

“-”: no available data.

**Abbreviation**

NIDSS: The Notifiable Infectious Diseases Surveillance System GP: General Population

BSTS: The Bayesian structural time series (BSTS) model PKA: the Robert Koch Institute, a national public health institute

SFOPH: The Swiss Federal Office of Public Health NDSRS: The National Disease Surveillance report system

WIDB: The Weekly Infectious Diseases Bulletin RDD: Regression Discontinuity Design

CPRC: The Crisis Preparedness and Responses Centre  DOCW: Dengue Operations Centre website

NBR: Negative Binomial Regression MCO: The Movement Control Order

PAF: The Partial Autocorrelation Function. CDC: The National Center for Epidemiology, Prevention and Disease Control

IRR: Incidence Rate Ratio SARIMA : Seasonal Autoregressive Integrated Moving Average

DSS: Dengue Surveillance System EUMH: The Epidemiology Unit of the Ministry of Health

ITS: Two-staged Interrupted Time Series NCDSS: The National Communicable Disease Surveillance System

PSM: Propensity Score Matching DID: A difference-in-difference (DID) identification strategy

SD: Social distance GLM: Generalized Linear Model

ESC: Epidemiological Surveillance Center RMNBR: Regression Models with Negative Binomial Response

NNDSS: Nationally Notifiable Diseases Surveillance System

PR: Poisson Regression WPRO IRIS: WHO Regional Office for the Western Pacific’s Institutional Repository for Information Sharing BRM: Bayesian Regression Model

MW: Migrant Worker GW: General Worker

MCGM: The Epidemiological Cell of Municipal Corporation of Greater Mumbai

HUMANITARIAN DATA EXCHANGE

**References**

[1] Xiao J, Dai J, Hu J, Liu T, Gong D, Li X, et al. (2021). Co-benefits of nonpharmaceutical intervention against COVID-19 on infectious diseases in China: A large population-based observational study. Lancet Reg Health West Pac. 17:100282. doi.org/10.1016/j.lanwpc.2021.100282

[2] Ullrich A, Schranz M, Rexroth U, Hamouda O, Schaade L, Diercke M, et al.(2021). Infectious Disease Surveillance Group. Impact of the COVID-19 pandemic and associated non-pharmaceutical interventions on other notifiable infectious diseases in Germany: An analysis of national surveillance data during week 1-2016 - week 32-2020. Lancet Reg Health Eur. 19;6:100103. doi: 10.1016/j.lanepe.2021.100103

[3] Steffen R, Lautenschlager S, Fehr J. (2020).Travel restrictions and lockdown during the COVID-19 pandemic-impact on notified infectious diseases in Switzerland. J Travel Med. 23;27(8):taaa180. doi: 10.1093/jtm/taaa180.

[5] Rahim MH, Dom NC, Ismail SNS, Mulud ZA, Abdullah S, Pradhan B. The impact of novel coronavirus (2019-nCoV) pandemic movement control order (MCO) on dengue cases in Peninsular Malaysia. One Health. 2021 Jan 29;12:100222.

[6] Plasencia-Dueñas, R., Failoc-Rojas, V. E., & Rodriguez-Morales, A. J. (2022). Impact of the COVID-19 pandemic on the incidence of dengue fever in Peru. Journal of medical virology, 94(1), 393–398. doi: org/10.1002/jmv.27298

[7] Ong, S. Q., Ahmad, H., & Mohd Ngesom, A. M. (2021). Implications of the COVID-19 Lockdown on Dengue Transmission in Malaysia. Infectious disease reports, 13(1), 148–160. doi: org/10.3390/idr13010016

[8] Niriella, M. A., Ediriweera, D. S., De Silva, A. P., Premarathna, B., Jayasinghe, S., & de Silva, H. J. (2021). Dengue and leptospirosis infection during the coronavirus 2019 outbreak in Sri Lanka. Transactions of the Royal Society of Tropical Medicine and Hygiene, 115(9), 944–946. doi: org/10.1093/trstmh/trab058

[9] Liyanage, P., Rocklöv, J., & Tissera, H. A. (2021). The impact of COVID-19 lockdown on dengue transmission in Sri Lanka; A natural experiment for understanding the influence of human mobility. PLoS neglected tropical diseases, 15(6), e0009420. doi: org/10.1371/journal.pntd.0009420

[10] Lim, J. T., Dickens, B. L., Ong, J., Aik, J., Lee, V. J., Cook, A. R., & Ng, L. C. (2021). Decreased dengue transmission in migrant worker populations in Singapore attributable to SARS-CoV-2 quarantine measures. Journal of travel medicine, 28(2), taaa228. doi: org/10.1093/jtm/taaa228

[11] Lim, J. T., Chew, L. Z. X., Choo, E. L. W., Dickens, B. S. L., Ong, J., Aik, J., . . . Cook, A. R. (2021). Increased Dengue Transmissions in Singapore Attributable to SARS-CoV-2 Social Distancing Measures. J Infect Dis, 223(3), 399-402. doi:10.1093/infdis/jiaa619.

[12] Li, N., Feng, Y., Vrancken, B., Chen, Y., Dong, L., Yang, Q., . . . Tian, H. (2021). Assessing the impact of COVID-19 border restrictions on dengue transmission in Yunnan Province, China: an observational epidemiological and phylogenetic analysis. Lancet Reg Health West Pac, 14, 100259. doi:10.1016/j.lanwpc.2021.100259.

[13] Conceição GMS, Barbosa GL, Lorenz C, et al. Effect of social isolation in dengue cases in the state of Sao Paulo, Brazil: An analysis during the COVID-19 pandemic. Travel Med Infect Dis. 2021;44:102149. doi:10.1016/j.tmaid.2021.102149.

[14] Bright A, Glynn-Robinson AJ, Kane S, Wright R, Saul N. The effect of COVID-19 public health measures on nationally notifiable diseases in Australia: preliminary analysis. Commun Dis Intell (2018). 2020;44:10.33321/cdi.2020.44.85. Published 2020 Oct 5. doi:10.33321/cdi.2020.44.85.

[15] Lai CC, Chen SY, Yen MY, Lee PI, Ko WC, Hsueh PR. The impact of the coronavirus disease 2019 epidemic on notifiable infectious diseases in Taiwan: A database analysis. Travel Med Infect Dis. 2021;40:101997. doi:10.1016/j.tmaid.2021.101997.

[16] Chen, Y., Li, N., Lourenço, J., Wang, L., Cazelles, B., Dong, L., Li, B., Liu, Y., Jit, M., Bosse, N. I., Abbott, S., Velayudhan, R., Wilder-Smith, A., Tian, H., Brady, O. J., & CMMID COVID-19 Working Group (2022). Measuring the effects of COVID-19-related disruption on dengue transmission in southeast Asia and Latin America: a statistical modelling study. The Lancet. Infectious diseases, S1473-3099(22)00025-1. Advance online publication. doi.org/10.1016/S1473-3099(22)00025-1

[17] Lu, X., Bambrick, H., Pongsumpun, P., Dhewantara, P. W., Toan, D., & Hu, W. (2021). Dengue outbreaks in the COVID-19 era: Alarm raised for Asia. PLoS neglected tropical diseases, 15(10), e0009778. doi.org/10.1371/journal.pntd.0009778
