# Supplementary material for: Effects of COVID-19 Non-Pharmacological Interventions on Dengue Infection: A Systematic Review and Meta-Analysis
Source: Front Cell Infect Microbiol. 2022 May 19;12:892508. doi: 10.3389/fcimb.2022.892508 (PMC9162155; doi:10.3389/fcimb.2022.892508)
Supplement: Supplementary file 10 [file Table_4.docx]

| Country  (Region) | 2015 | 2016 | 2017 | 2018 | 2019 | 2020 | 2021 | Average  (2015-2019) | Change Rate  (2020) | Change Rate  (2021) |
| --- | --- | --- | --- | --- | --- | --- | --- | --- | --- | --- |
| Malaysia | 111285 | 100028 | 82840 | 80615 | 127407 | 88074 | 24589 | 100435 | -0.1231 | -0.7552 |
| Thailand | 84600 | 29000 | 51000 | 54482 | 86418 | 50042 | 7180 | 61100 | -0.1810 | -0.8825 |
| Sri Lanka | 29777 | 55150 | 186101 | 51659 | 105049 | 31162 | 19639 | 85547 | -0.6357 | -0.7704 |
| Philippines | 169435 | 176411 | 144915 | 199271 | 437563 | 83155 | 66655 | 225519 | -0.6313 | -0.7044 |
| Singapore | 11298 | 13051 | 2689 | 3126 | 15622 | 35356 | 4991 | 9157 | 2.8610 | -0.4550 |
| Cambodia | 15412 | - | 3195 | 9885 | 65000 | 11977 | 1523 | 23373 | -0.4876 | -0.9348 |
| Lao PDR | 1952 | 5373 | 11039 | 6204 | 38753 | 7554 | 1328 | 12664 | -0.4035 | -0.8951 |
| Viet Nam | 79912 | 79204 | 183287 | 113850 | 320702 | 121398 | 68268 | 155391 | -0.2188 | -0.5607 |
| Brazil | 1649008 | 1500535 | 252054 | 265934 | 2248570 | 1467142 | 975474 | 1183220 | 0.2400 | -0.1756 |
| Colombia | 96444 | 101016 | 25284 | 44825 | 127553 | 78979 | 53334 | 79024 | -0.0006 | -0.3251 |
| Mexico | 219593 | 130069 | 89893 | 78621 | 268458 | 120639 | 36742 | 157327 | -0.2332 | -0.7665 |
| Peru | 35837 | 31868 | 76093 | 6930 | 17143 | 56394 | 49274 | 33574 | 0.6797 | 0.4676 |
| Argentina | 4774 | 79455 | 557 | 1829 | 3220 | 59358 | 3972 | 17967 | 2.3037 | -0.7789 |
| Bolivia | 27099 | 32386 | 9239 | 7727 | 19987 | 111347 | 8947 | 19288 | 4.7730 | -0.5361 |
| Ecuador | 42499 | 13612 | 11387 | 3094 | 8416 | 16570 | 20592 | 15802 | 0.0486 | 0.3032 |
| Honduras | 44834 | 22961 | 5217 | 7942 | 132143 | 25180 | 19753 | 42619 | -0.4092 | -0.5365 |
| Nicaragua | 49326 | 88463 | 64712 | 58746 | 186173 | 53953 | 36741 | 89484 | -0.3971 | -0.5894 |
| Paraguay | 68652 | 70215 | 1832 | 32359 | 11811 | 223782 | 16897 | 36974 | 5.0524 | -0.5430 |
| Venezuela | 54152 | 29268 | 8615 | 19118 | 16015 | 6721 | 5926 | 25434 | -0.7357 | -0.7670 |
| Belize | 5691 | 4899 | 3042 | 2326 | 13316 | 2785 | 1251 | 5855 | -0.5243 | -0.7863 |
| Total | 2803595 | 2588353 | 1215008 | 1050561 | 4249319 | 2651568 | 1423076 | 2381367 | 0.1135 | -0.4024 |
| Americas | 2416018 | 2175409 | 579027 | 561689 | 3190778 | 2326115 | 1254648 | 1784584 | 0.3034 | -0.2970 |

Supplementary Table 4. Notified cases of dengue fever from 2015-2021 in most highly dengue-endemic countries in Asia and Americas
