# Supplementary material for: Effects of COVID-19 Non-Pharmacological Interventions on Dengue Infection: A Systematic Review and Meta-Analysis
Source: Front Cell Infect Microbiol. 2022 May 19;12:892508. doi: 10.3389/fcimb.2022.892508 (PMC9162155; doi:10.3389/fcimb.2022.892508)
Supplement: Supplementary file 11 [file Table_5.docx]

Supplementary Table 5. Stratified analysis for the effect of NPIs on dengue infection

based on the emergency response level.

| Study | Emergency response /corrective action and duration(the reduction rate of dengue infection) | | |
| --- | --- | --- | --- |
|  | Level 1^a^ | Level 2^b^ | Level 3^c^ |
| [1] | Weeks 4-8 (91.7%) | Weeks 9-19 (97.2%) | Weeks 20-53 (99.4%) |
| [2] | Weeks 11-17(reduction) | Weeks 18-31(significant reduction) | |

a : lockdown, travel restrictions, crowd prohibition, and compulsory health quarantine.

b : During this period, residents from or with a history of travel to overseas countries and territories needed to be quarantined for 14 days and receive health management. International flights were subsequently cut down, and travel was restricted to a specific airline.

c: During this period, schools returned to a normal routine, but travel-related and case-based NPIs continued to be implemented. However, some NPIs had been alleviated.

[1] Xiao J, Dai J, Hu J, Liu T, Gong D, Li X, et al. (2021). Co-benefits of nonpharmaceutical intervention against COVID-19 on infectious diseases in China: A large population-based observational study. Lancet Reg Health West Pac. 17:100282. doi.org/10.1016/j.lanwpc.2021.100282

[2] Ullrich A, Schranz M, Rexroth U, Hamouda O, Schaade L, Diercke M, et al.(2021). Infectious Disease Surveillance Group. Impact of the COVID-19 pandemic and associated non-pharmaceutical interventions on other notifiable infectious diseases in Germany: An analysis of national surveillance data during week 1-2016 - week 32-2020. Lancet Reg Health Eur. 19;6:100103. doi: 10.1016/j.lanepe.2021.100103
